# Acetylation-triggered degradation of MSX1 impairs palatal development

**DOI:** 10.1038/s41420-026-03018-w

**Published:** 2026-03-19

**Authors:** Li Meng, Jiawen You, Zhongyin Zhang, Yucheng Jiang, Yulan Liu, Mingliang Zhou, Junqing Ma, Xinquan Jiang

**Affiliations:** 1 Department of Prosthodontics, Shanghai Ninth People’ s Hospital, Shanghai Jiao Tong University School of Medicine, College of Stomatology, Shanghai Jiao Tong University, National Center for Stomatology, National Clinical Research Center for Oral Diseases, Shanghai Key Laboratory of Stomatology, Shanghai Engineering Research Center of Advanced Dental Technology and Materials, Shanghai, China; 2 Stomatological Hospital affiliated Suzhou Vocational Health College, Suzhou, China; 3 State Key Laboratory Cultivation Base of Research, Prevention and Treatment for Oral Diseases, Nanjing Medical University, Nanjing, China; 4 Shanghai Stomatological Hospital, Fudan University, Shanghai, China; 5 Savaid Stomatology School, Hangzhou Medical College, Hangzhou, China; 6 Hangzhou Stomatological Hospital (Zijingang Campus), Hangzhou, China

**Keywords:** Epigenetics, Development, Proteolysis, Gene therapy, Cell growth

## Abstract

Cleft palate, a prevalent congenital disorder, arises from dysregulated embryonic palatal fusion, but the posttranslational modifications (PTMs) driving this process remain poorly understood. Here, we report that lysine acetylation is a critical MSX1 proteostasis switch that governs embryonic palatal mesenchymal (EPM) cell survival. We demonstrate in vitro and in vivo that MSX1 protein stability regulation by deacetylase SIRT1-catalyzed acetylation underlies EPM apoptosis and palatal fusion. In atRA-induced cleft palate models, SIRT1 suppression drives MSX1 hyperacetylation, accelerating proteasomal degradation and culminating in EPM apoptosis. Strikingly, transcriptomic profiling revealed the exclusive proteostatic role of acetylation, indicating that MSX1’s structural stability differs from its transcriptional activity—a paradigm distinct from that of classic PTM mechanisms during development. Lentivirus-mediated delivery of the deacetylase SIRT1 or the deacetylation mimic MSX1 K139R significantly reduced cleft severity, indicating its preventive and therapeutic potential in humans. Our work establishes the MSX1 acetylation as both a pathogenic driver and a druggable target in cleft palate, redefining PTM regulation as a central etiological factor in genetic disorders.

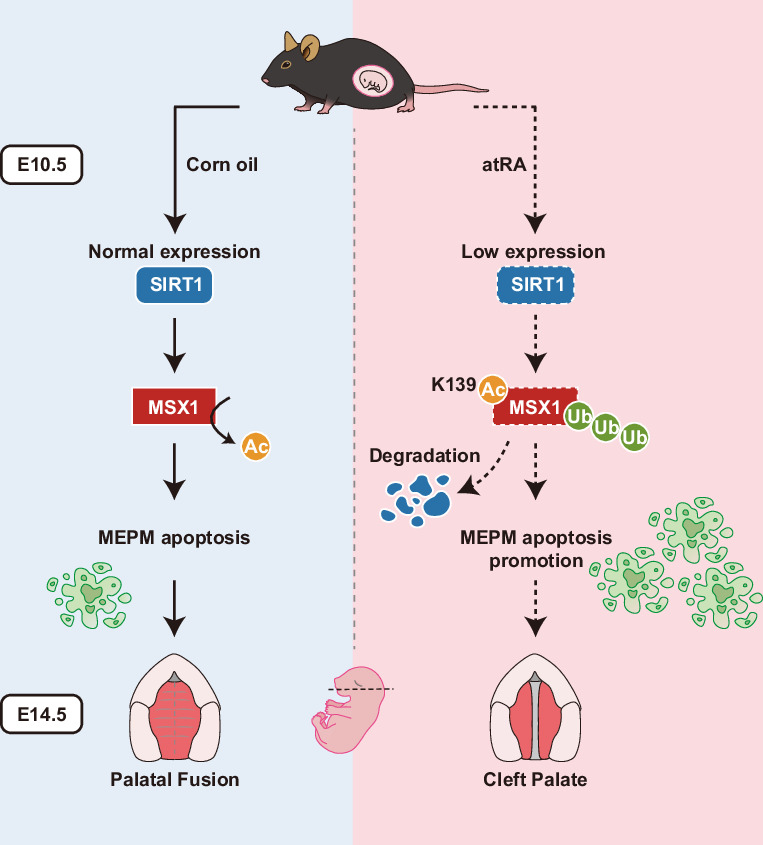

## Introduction

Cleft palate, one of the most prevalent congenital craniofacial defects, substantially affects neonatal respiratory function, oropharyngeal development, and long-term psychosocial well-being [1]. It occurs in approximately 1–25 cases per 10,000 births worldwide [2]. This developmental anomaly arises from the disruption of palatal fusion, a precisely orchestrated morphogenetic process requiring spatiotemporal coordination of EPM cell migration, proliferation, and apoptosis [3]. While both genetic factors and environmental teratogens contribute to cleft palate pathogenesis [4], recent advances have indicated that the dysregulation of key morphogenetic regulators is central to disease etiology [5].

Muscle segment homeobox 1 (MSX1) has emerged as a pivotal regulator of craniofacial morphogenesis through transcriptional control of epithelial‒mesenchymal interactions [5, 6]. Genetic studies in humans have identified more than 10 pathogenic MSX1 variants, accounting for 2% of all cleft palate cases [7–9]. Msx1^-/-^ embryos exhibit a complete cleft secondary palate with concomitant tooth bud arrest and defective cranial neural crest differentiation [6]. Spatiotemporal expression analyses revealed the dynamic localization of Msx1 in the anterior palatal mesenchyme during critical fusion windows (E12.5–E14.5) [5, 10], indicating that Msx1 is a master regulator of palatogenesis. Despite these advances, the posttranslational regulatory mechanisms that fine-tune MSX1 activity during palatal development remain unknown.

Posttranslational modifications (PTMs) have recently emerged as critical modulators of homeodomain transcription factor function. While MSX1 phosphorylation modulates limb patterning through BMP signaling [11] and SUMOylation regulates its nucleocytoplasmic shuttling [12–14], the functional importance of lysine acetylation, a ubiquitous PTM that governs transcriptional complex assembly [15], remains unexplored. This knowledge gap is particularly intriguing given the physical interaction of MSX1 with CBP/p300 acetyltransferases [16], which are enzymatic mediators of 2/3 nuclear acetylation events [17]. We hypothesize that dynamic acetylation‒deacetylation cycles may critically regulate MSX1 protein stability and transcriptional repressor activity during palatal morphogenesis.

Here, we identify MSX1 deacetylation as a novel regulatory process controlling MSX1 stability in EPM cells using an all-trans retinoic acid (atRA)-induced cleft palate model. Through site-directed mutagenesis and protein functional assays, we demonstrated that acetylation at lysine 139 (K139) triggers ubiquitin–proteasomal degradation of MSX1, resulting in accelerated EPM apoptosis and failure of palatal shelf fusion. Crucially, pharmacological SIRT1 activation or acetylation-resistant MSX1 K139R mutation significantly rescued atRA-induced palatal defects. These findings establish MSX1 acetylation dynamics as a molecular nexus integrating genetic susceptibility (MSX1 variants) and environmental teratogenesis (atRA exposure), not only elucidating a novel PTM regulatory mechanism underlying cleft palate pathogenesis but also revealing pharmacologically targetable pathways for prevention.

## Results

### AtRA induces cleft palate through aberrant MEPM apoptosis via MSX1 acetylation

**Fig. 1:**
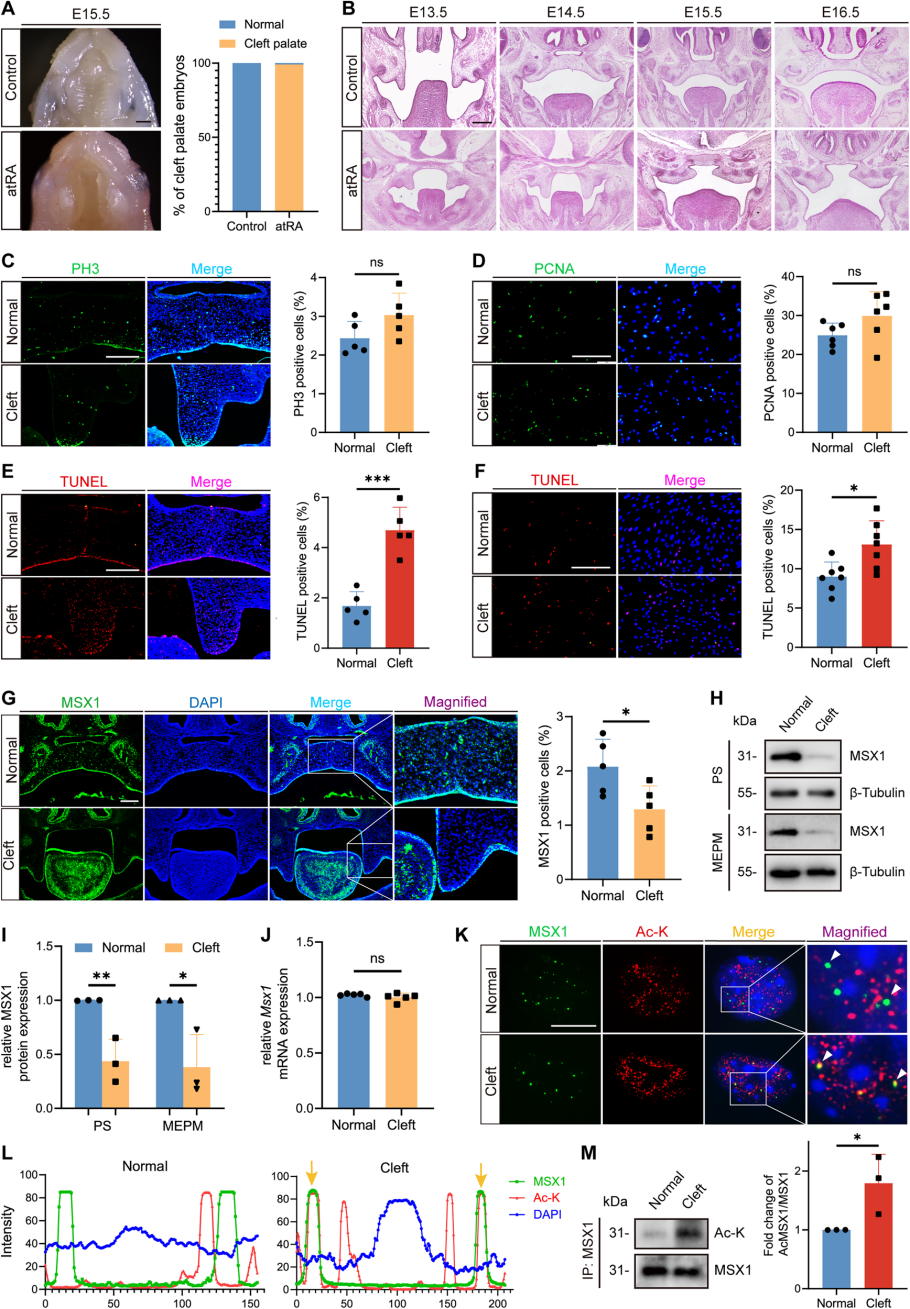
AtRA induces cleft palate through aberrant MEPM cell apoptosis and MSX1 acetylation. **A** Ventral view of the palatal shelves in Control and atRA treated mice at E15.5 and quantification of cleft palate rate of embryos. Bars, 200 µm. *n* = 115. **B** HE staining of palatal shelves from E13.5 to E16.5. Bars, 200 µm. **C** PH3 immunostaining (green) of palatal shelves from E14.5 embryos and quantitative analysis. Bars, 200 µm. *n* = 5. **D** PCNA immunostaining (green) of MEPM cells extracted from E14.5 embryos and quantitative analysis. Bars, 200 µm. *n* = 6. **E** TUNEL staining (red) of palatal shelves from E14.5 embryos and quantitative analysis. Bars, 200 µm. *n* = 5. **F** TUNEL staining (red) of MEPM cells extracted from E14.5 embryos and quantitative analysis. Bars, 200 µm. *n* = 7. **G** Immunofluorescent staining of MSX1 (green) of palatal shelves in E14.5 mice, and quantification of MSX1 positive cells. Bars, 200 µm. *n* = 5. **H** Western blot analysis of protein expression of MSX1 in palatal shelves and MEPM cells extracted from embryos. **I** Quantification of MSX1 protein level in palatal shelves and MEPM cells extracted from embryos. *n* = 3. **J** qRT-PCR analysis of mRNA expression of *Msx1* in total RNA extracted from palatal shelves of E14.5 embryos. *n* = 5. **K** Immunofluorescence staining of MSX1 (green) and Ac-lysine (red) in MEPM cells extracted from embryos. The white arrows indicate colocalization. Bars, 10 µm. **L** Colocalization analysis of MSX1 and Ac-K. **M** Acetylation of endogenous MSX1 in palatal shelf tissues from embryos by IP assay, and quantification of acetylated MSX1 protein level. *n* = 3. PS, palatal shelves. Ac-K, Ac-lysine. Data are presented as the mean ± standard deviation (Mean ± SD). ns, not significant, *p < 0.05, **p < 0.01, ***p < 0.001.

To investigate the pathogenic mechanism of cleft palate, we established an atRA (100 mg/kg)-induced cleft palate model by administering atRA to pregnant mice by gavage on embryonic day 10.5 (E10.5) [18], which resulted in cleft palate in 99.13% of cases by E15.5 (Fig. 1A). Histological analysis revealed no elevation of the bilateral palatal shelves at E14.5, and the elevated palate shelves were smaller in the atRA group than in the control group and thus failed to attach to the midline at E15.5 (Fig. 1B).

Strikingly, functional characterization of MEPM cells revealed that while proliferation was comparable between the groups (Fig. 1C, D), apoptosis was markedly increased in cleft palate, both in vivo (Fig. 1E) and in primary MEPM cell cultures (Fig. 1F; Supplementary Fig. 1A).

To determine the role of MSX1 in the regulation of MEPM cell apoptosis, we analyzed MSX1 expression and detected significantly reduced protein levels in the cleft palate group (Fig. 1G–I) despite the lack of change in mRNA levels (Fig. 1J), suggesting the possibility that protein stability is regulated via PTMs. Short hairpin RNA (shRNA)-mediated MSX1 knockdown in MEPM cells recapitulated the apoptotic phenotype (Supplementary Fig. 1B–E), indicating that MSX1 protein stability is critical for suppressing MEPM cell apoptosis and promoting palate development.

Given that global lysine acetylation increased by more than 1.56- and 1.6-fold at E13.5 and E14.5, respectively, in cleft palate (Supplementary Fig. 1F) and that MSX1 is known to interact with the acetyltransferase CBP/p300 [19, 20], we hypothesized that acetylation-mediated destabilization occurred. Immunofluorescence confirmed the increase in the colocalization of MSX1 with acetylated lysine (Ac-K) in cleft palate (Fig. 1K, L), whereas immunoprecipitation (IP) assays revealed a 1.8-fold increase in MSX1 acetylation (Fig. 1M).

Collectively, these findings demonstrate that atRA-induced cleft palate results from MSX1 acetylation-mediated protein destabilization, leading to excessive MEPM cell apoptosis.

### MSX1 is deacetylated by SIRT1 at K139

**Fig. 2:**
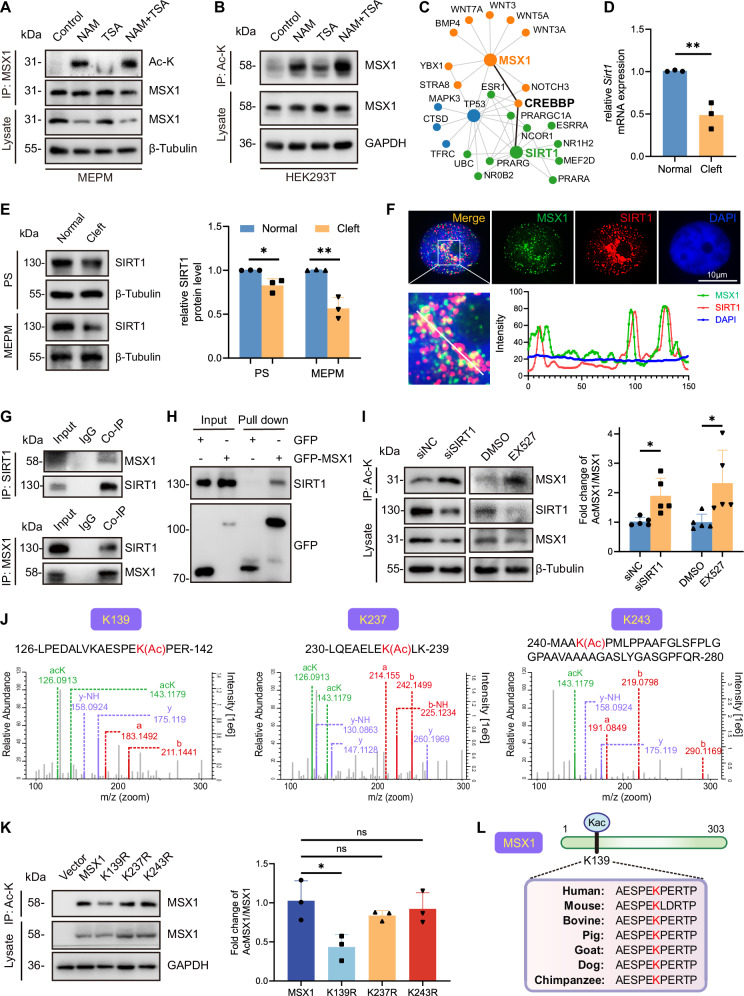
MSX1 is deacetylated by SIRT1 at K139. **A** Acetylation of endogenous MSX1 in MEPM cells treated with 500 nM TSA or 20 mM NAM for 6 h by IP assay. **B** Acetylation of exogenous GFP-MSX1 in HEK293T cells treated with 500 nM TSA or 20 mM NAM for 6 h by IP assay. **C** Bioinformatic analyses of potential interacting proteins of MSX1. **D** qRT-PCR analysis of mRNA expression of *Sirt1* in total RNA extracted from palatal shelves of E14.5 embryos. **E** Western blot analysis of protein expression of SIRT1 in palatal shelves and MEPM cells respectively extracted from embryos. *n* = 3. **F** Immunofluorescence staining of MSX1 (green) and SIRT1 (red) in MEPM cells, and colocalization analysis of MSX1 and SIRT1. **G** Co-IP analysis of MSX1 with SIRT1. **H** In vitro GFP pulldown analysis of MSX1 and SIRT1. **I** IP analysis of MSX1 acetylation level in MEPM cells transfected with normal control siRNA (siNC) or SIRT1 siRNA (siSIRT1), or treated with DMSO or 10 µM EX527 for 24 h, followed by quantification analysis, *n* = 5. **J** Potential acetylation sites in MSX1 analyzed by mass spectrometry. **K** IP analysis of MSX1 acetylation level in MEPM cells transfected with Vector, full-length GFP-MSX1 or K139R, K237R, K243R mutants, and quantification analysis. *n* = 3. **L** Alignment of MSX1 protein sequence across different species. TSA, Trichostatin A. NAM, Nicotinamide. PS, palatal shelves. Ac-K, Ac-lysine. Data are presented as the mean ± standard deviation (Mean ± SD). ns not significant, *p < 0.05, **p < 0.01.

To elucidate the regulatory mechanism of MSX1 acetylation, we first validated its acetylation dynamics in both endogenous and exogenous contexts. Treatment of primary MEPM cells with the deacetylase inhibitors NAM (a SIRT family inhibitor) and TSA (an HDAC inhibitor) markedly increased MSX1 acetylation, with NAM having a stronger effect, and this change was accompanied by reduced protein levels (Fig. 2A). Similarly, the acetylation of exogenous MSX1-transfected 293 T cells increased upon NAM treatment (Fig. 2B), indicating the regulation of conserved acetylation.

The STRING database was used to predict interactions among MSX1, the acetyltransferase CBP, which mediates more than 2/3 of the known types of lysine acetylation [17], and the deacetylase SIRT1, the most extensively studied member of the SIRT family [21] (Fig. 2C). Although MSX1 colocalized and interacted with CBP/p300 in MEPM cells (Supplementary Fig. 2A–C), its expression remained unaltered in cleft palate (Supplementary Fig. 2D, E), suggesting that pathological MSX1 hyperacetylation might stem from defective deacetylase activity. Notably, both SIRT1 mRNA levels and SIRT1 protein levels were significantly reduced in cleft palate tissues, with concordant downregulation in primary MEPM cells (Fig. 2D, E, Supplementary Fig. 2F).

Given that (1) NAM targets SIRT family members, (2) MSX1 and SIRT1 are predicted to interact, (3) SIRT1 is downregulated in cleft palate, and (4) Sirt1-null mice exhibit palatal defects [22], we hypothesized that SIRT1 plays an important role in regulating palatal development through MSX1 acetylation. Mechanistically, immunofluorescence revealed the nuclear colocalization of MSX1 and SIRT1 in the nuclei of MEPM cells (Fig. 2F), whereas coimmunoprecipitation (co-IP) confirmed the interaction between MSX1 and SIRT1 (Fig. 2G); moreover, GFP pulldown with the purified MSX1 protein confirmed direct binding (Fig. 2H, Supplementary Fig. 2G). Deacetylase inhibition with siSIRT1 or SIRT1 inhibition with EX527 significantly increased MSX1 acetylation and accelerated protein degradation, whereas SIRT1 knockdown did not significantly affect the mRNA level of MSX1 (Fig. 2I, Supplementary Fig. 2H–J), indicating that SIRT1 is the dominant deacetylase.

Mass spectrometry revealed three putative acetylation sites, K139, K237 and K243 (Fig. 2J), in MSX1, with K139 being the dominant site, as evidenced by the abolished acetylation of K139R mutants (Fig. 2K). To further confirm this finding, we generated a series of GFP-tagged MSX1 truncation mutants (Δ43-79, Δ79-172, and Δ172-239) (Supplementary Fig. 3A). Co-IP assays in which these mutants were coexpressed with Flag-SIRT1 revealed that compared with the other trunction mutants and MSX1 wild type, the Δ79-172 mutant, which lacks a region containing K139, strongly weakened the interaction with SIRT1 compared to the other truncation mutants and MSX1 wild type (Supplementary Fig. 3B). The results of the GFP pulldown assay consistently demonstrated that the binding of the Δ79-172 mutant to SIRT1 was significantly reduced (Supplementary Fig. 3C), confirming that SIRT1 interacts with MSX1 specifically within the region spanning residues 79-172, which contains the identified acetylation site K139. In addition, evolutionary analysis revealed strong conservation of the K139 residue across vertebrates, suggesting its critical functional preservation (Fig. 2L).

These findings indicate that SIRT1-mediated deacetylation at K139 is an essential regulatory switch that controls MSX1 protein homeostasis during palatogenesis.

### MSX1 lysine acetylation drives MEPM cell apoptosis

**Fig. 3:**
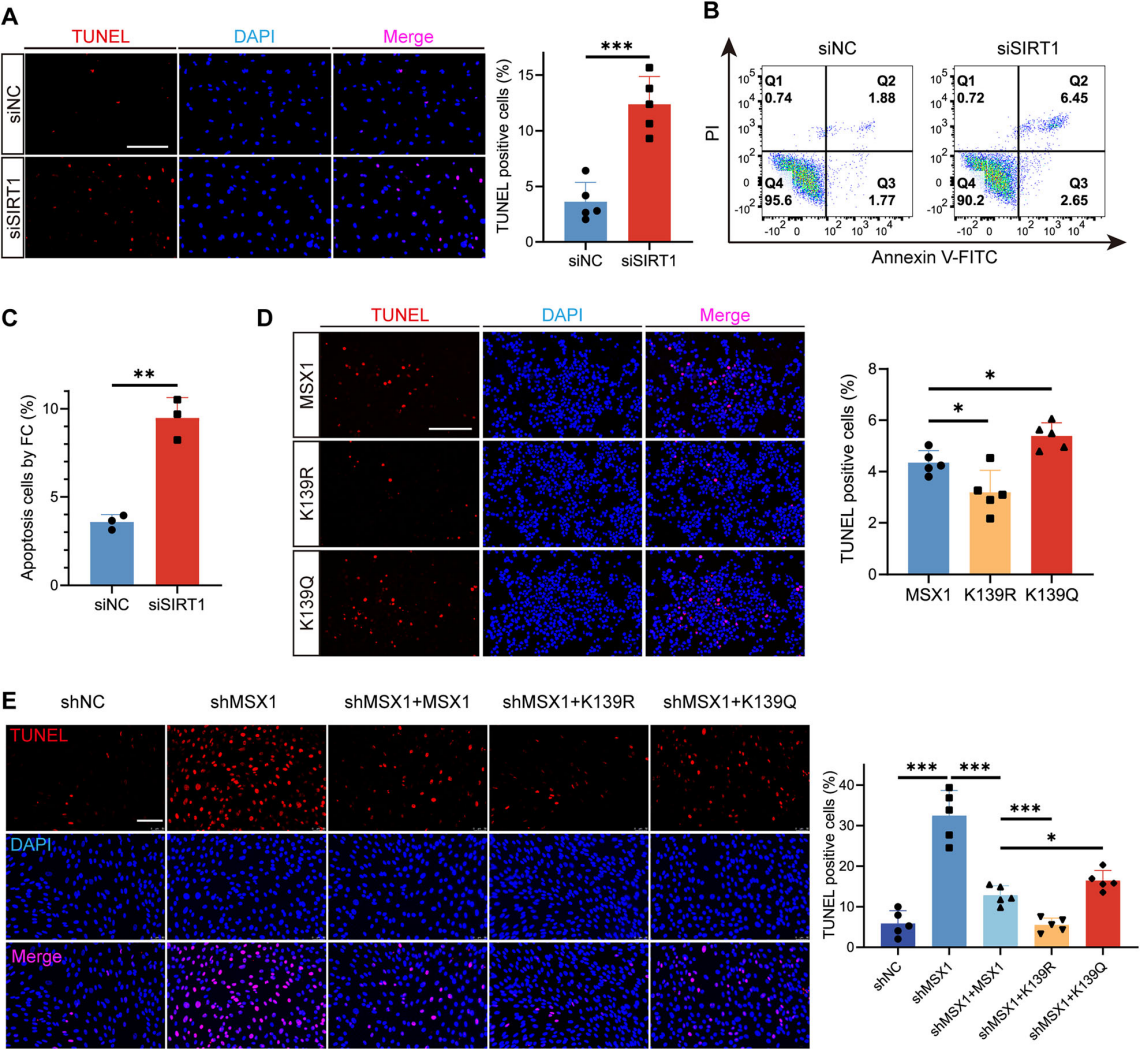
MSX1 lysine acetylation drives MEPM apoptosis. **A** TUNEL staining (red) of MEPM cells transfected with siNC or siSIRT1. Bars, 200 µm. *n* = 5. **B** Flow cytometry analysis of cell apoptosis in MEPM cells transfected with siNC or siSIRT1. **C** Quantitative analysis of flow cytometry. *n* = 3. **D** TUNEL staining (red) of MEPM cells transfected with MSX1 full-length (MSX1), K139R and K139Q mutants. **E** TUNEL staining (red) of MEPM cells transfected with normal control shRNA (shNC), MSX1 shRNA (shMSX1) and combinations of shMSX1 and MSX1, K139R or K139Q mutants. Bars, 200 µm. *n* = 5. Data are presented as the mean ± standard deviation (Mean ± SD). *p < 0.05, **p < 0.01, ***p < 0.001.

To investigate the functional impact of MSX1 acetylation on MEPM apoptosis, we genetically modulated SIRT1 expression and mimicked MSX1 acetylation. SIRT1 knockdown in MEPM cells increased the percentage of TUNEL-positive cells (Fig. 3A), and flow cytometry confirmed a 2.6-fold increase in the number of apoptotic cells (Fig. 3B, C), indicating that SIRT1 deficiency recapitulates the apoptotic phenotype observed in the cleft palate model.

We next directly tested the functional consequences of MSX1 acetylation by generating K139R (deacetylation mimic) and K139Q (acetylation mimic) mutants. Compared with wild-type MSX1, K139R reduced the percentage of TUNEL-positive cells to 3.19 ± 0.86% (vs. WT: 4.65 ± 0.26%), whereas K139Q increased the percentage of apoptotic cells to 5.26 ± 0.70% (Fig. 3D). These results demonstrate that the acetylation status of K139 is sufficient to modulate MEPM cell survival.

To definitively establish the functional hierarchy of K139 acetylation independent of endogenous MSX1, we performed a knockdown and rescue experiment. Depletion of endogenous MSX1 expression significantly increased apoptosis, and re‑expression of wild‑type MSX1 partially restored cell survival. Strikingly, the K139R mutant provided the strongest rescue, significantly outperforming wild‑type MSX1, while the K139Q mutant showed only a partial rescue effect (Fig. 3E). This genetic epistasis experiment in a clean background confirms that the acetylation status at K139 is a primary determinant of the antiapoptotic function of MSX1.

To elucidate the mechanism through which MSX1 regulates apoptosis, we then performed qRT–PCR in MSX1-knockdown cells (shMSX1). Consistent with a pro-survival role, MSX1 depletion led to a significant downregulation of the antiapoptotic gene Bcl2 and upregulation of the proapoptotic gene Caspase 3. The expression of Bax and P21 lacked significant change (Supplementary Fig. 4A). These findings identify Bcl2 and Caspase 3 as direct or indirect targets of MSX1 in the palatal mesenchyme, providing a molecular explanation for the apoptotic phenotype observed upon MSX1 loss or destabilization.

Together, these data demonstrate that SIRT1 maintains MEPM cell survival by restraining MSX1 acetylation at K139, and that hyperacetylation of this residue drives MEPM apoptosis, indicating that MSX1 K139 is a critical functional switch in palatal cell fate determination.

### Lysine acetylation of MSX1 promotes proteasomal degradation

**Fig. 4:**
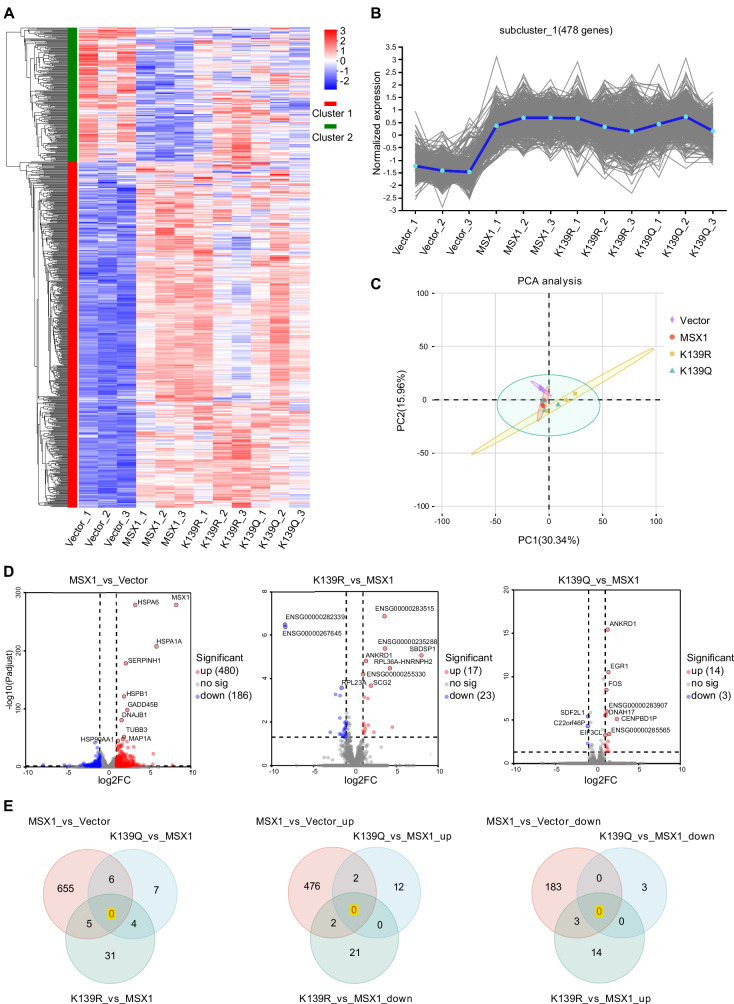
Lysine acetylation of MSX1 promotes proteasomal degradation. **A** Cluster heatmap of RNA-seq data from MEPM cells transfected with Vector, MSX1, K139R or K139Q plasmids. Red represents upregulation, and blue represents downregulation. **B** Trend plots of the largest subcluster (478 genes). **C** Principle component analysis (PCA) analysis of transcriptome data from twelve samples. **D** Volcano plots of differentially expressed genes (DEGs) (*P* ≤ 0.05, |log2FC | ≥ 2). **E** Venn diagram analysis of DEGs.

To determine the functional impact of MSX1 acetylation, we integrated transcriptomic profiling and protein stability assays. RNA-seq of MEPM cells transfected with an empty vector, MSX1, K139R (deacetylation mimic), or K139Q (acetylation mimic) plasmid revealed a total of 38030 genes and 178770 transcripts (Fig. 4A). Gene expression clustering analysis and principal component analysis (PCA, PC1 = 30.34%, PC2 = 15.96%) revealed highly similar expression profiles among the MSX1, K139R and K139Q groups (Fig. 4A–C). Consistently, qRT‒PCR revealed identical expression patterns among genes related to critical palatogenesis pathways (the BMP, TGFβ, WNT/β-catenin, FG and SHH signaling pathways) (Supplementary Fig. 4B–F) [5, 23]. In addition, the expression levels of MSX1 transcriptional targets (Bmp2, Dlx2, Dlx5, Dlx6, MyoD1, P21, Twist1, Snai1, Snai2, and Bcl2) were not specifically altered by the modulation of MSX1 acetylation in the K139R and K139Q mutants (Supplementary Fig. 4G) [19, 20, 24–27], indicating that no global transcriptional shifts were caused by acetylation.

Analysis of differentially expressed genes (DEGs; *P* ≤ 0.05, |log2FC | ≥ 2) revealed 666 DEGs (480 upregulated, 186 downregulated) in the MSX1 group compared with the empty vector group, 40 DEGs (17 upregulated, 23 downregulated) in the K139R group compared with the MSX1 group and 17 DEGs (14 upregulated, 3 downregulated) in the K139Q group compared with the MSX1 group (Fig. 4D). Venn analysis revealed no intersection among these gene sets (Fig. 4E). GO and KEGG analyses of the DEGs revealed enrichment in pathways unrelated to the canonical role of MSX1 (e.g., mesenchymal stem cell proliferation), with no association with palatogenesis or apoptosis (Supplementary Fig. 5A–F), further corroborating the transcriptional inertness of MSX1 acetylation.

**Fig. 5:**
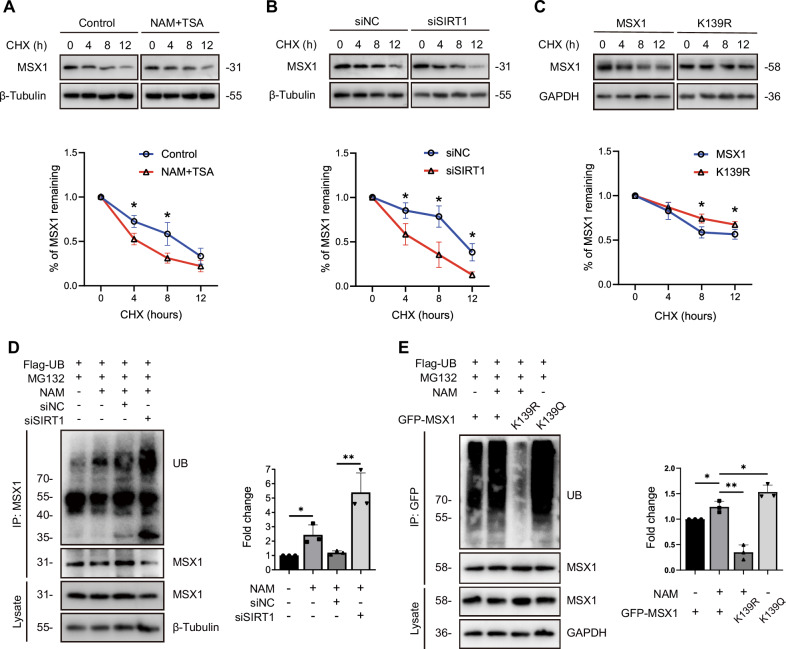
Lysine acetylation of MSX1 promotes proteasomal degradation. **A–C** MSX1 protein stability assessed by western blot analysis with cycloheximide (CHX) chase. **A** MSX1 protein stability in MEPM cells treated with or without 500 nM TSA and 20 mM NAM for 6 h. *n* = 3. **B** MSX1 protein stability in MEPM cells transfected with siNC or siSIRT1. *n* = 3. **C** MSX1 protein stability in MEPM cells transfected with MSX1 or K139R mutant. *n* = 3. **D,E** Ubiquitination of MSX1 measured by IP analysis with MG132. **D** Ubiquitination of MSX1 in MEPM cells transfected with siNC or siSIRT1 and quantitative analysis. **E** Ubiquitination of MSX1 in MEPM cells transfected with MSX1, K139R or K139Q mutants and quantitative analysis. *n* = 3. TSA, Trichostatin A. NAM, Nicotinamide. CHX, cycloheximide. Data are presented as the mean ± standard deviation (Mean ± SD). *p < 0.05, **p < 0.01.

Despite this transcriptional inertia, MSX1 protein dynamics exhibited striking acetylation dependency. Cycloheximide (CHX) chase assays revealed that enhanced MSX1 acetylation by deacetylase inhibition (NAM + TSA) or SIRT1 knockdown accelerated MSX1 protein degradation (Fig. 5A, B), whereas the K139R mutation slowed MSX1 protein degradation (Fig. 5C). This indicates that lysine acetylation has a detrimental effect on the stability of the MSX1 protein.

Mechanistically, ubiquitination assays revealed that SIRT1 inhibition increased MSX1 ubiquitination, the canonical proteasomal degradation signal, by 2.39-fold (Fig. 5D); however, the K139R mutation decreased ubiquitination by 30%, whereas the K139Q mutation increased it by 1.45-fold (Fig. 5E).

Taken together, these findings indicate that lysine acetylation destabilizes MSX1 via ubiquitin‒proteasome degradation without affecting its transcriptional function, establishing that its pathological role in cleft palate stems from protein destabilization rather than transcriptional dysregulation.

### Reduced MSX1 lysine acetylation partially rescues atRA-induced cleft palate

**Fig. 6:**
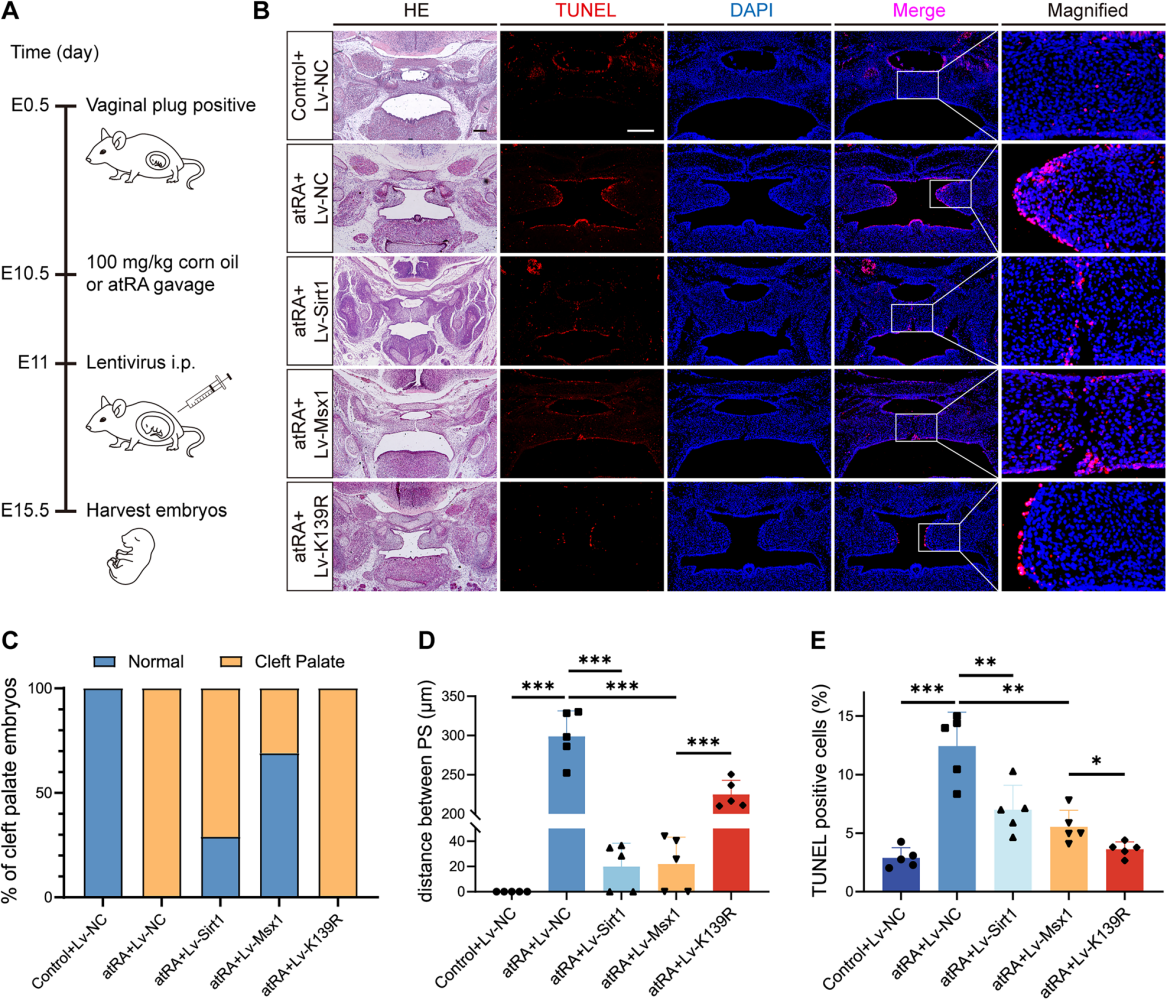
Reduced MSX1 lysine acetylation partially rescues atRA-induced cleft palate. **A** The protocol on the lentivirus transduction of mice. **B** HE and TUNEL staining (red) of coronal sections of murine palate at E15.5. Nuclei were stained with DAPI. Bars, 200 µm. **C** Statistics of cleft palate and normal mouse embryos. **D** Statistics of distance between bilateral palatal shelves of mouse embryos. *n* = 5. **E** Quantification of TUNEL assays of palatal shelves of mouse embryos. *n* = 5. I.p., intraperitoneal injection. Data are presented as the mean ± standard deviation (Mean ± SD). *p < 0.05, **p < 0.01, ***p < 0.001.

To evaluate the therapeutic potential of targeting MSX1 lysine acetylation, we designed a preclinical rescue paradigm combining atRA exposure with lentiviral delivery (1*10^9^ TU via intraperitoneal injection) of a negative control (Lv-NC), SIRT1 (Lv-SIRT1), wild-type MSX1 (Lv-MSX1) or the MSX1 deacetylation mimic K139R (Lv-K139R) at E10.5 (Fig. 6A). Immunofluorescence staining confirmed successful lentivirus delivery, in which SIRT1 protein expression increased 1.78-fold, whereas MSX1 and K139R expression increased 1.44- and 1.64-fold, respectively (Supplementary Fig. 6A‒C). HE staining revealed that the atRA+Lv-NC group presented a complete cleft palate (100% incidence) with a palatal gap of 0.82 ± 0.12 mm, whereas the control+Lv-NC group presented a palatal gap of 0.12 ± 0.03 mm. The atRA+Lv-Sirt1 and atRA+Lv-Msx1 groups presented nearly complete palatal fusion, with the incidence of cleft palate decreasing to 71% and 31%, respectively. Although the incidence of cleft palate did not decrease in the atRA+Lv-K139R group, the palatal gap was significantly reduced (Fig. 6B‒D). TUNEL assays revealed apoptosis rates of 12.94 ± 2.16% in the atRA+Lv-NC group and 3.20 ± 1.00% in the control+Lv-NC group; however, the apoptosis rate decreased to 3.67 ± 0.19% in the atRA+Lv-K139R group, to 6.68 ± 0.68% in the atRA+Lv-Sirt1 group and to 6.21 ± 1.44% in the atRA+Lv-Msx1 group (Fig. 6B, E).

Together, these findings indicate that reducing MSX1 acetylation via SIRT1 overexpression or the K139R mutation partially rescues atRA-induced cleft palate and decreases MEPM apoptosis, highlighting deacetylation as a potential therapeutic target for cleft palate.

## Discussion

### A PTM-centric paradigm of craniofacial morphogenesis

This study elucidated the central role of MSX1 lysine acetylation in cleft palate and the molecular mechanisms underlying this role through a multidimensional experimental approach (Graphical Abstract). In the atRA-induced cleft palate model, SIRT1 downregulation led to excessive MSX1-K139 acetylation, which increased MSX1-K139 ubiquitination and accelerated MSX1-K139 degradation, ultimately triggering MEPM cell apoptosis and failed fusion. Strikingly, this pathway operates independently of the transcriptional function of MSX1, as evidenced by transcriptomic inertia across acetylation states. Rescue experiments demonstrated that SIRT1 overexpression or the K139R mutation partially restored MSX1 stability, reduced apoptosis and decreased cleft incidence, validating the targeting of deacetylation as a viable therapeutic strategy. Our findings challenge the prevailing mutation-centric view of cleft palate pathogenesis, positioning dynamic PTM regulation as a key etiological determinant. This study is the first to link MSX1 acetylation directly to cleft palate and proposes the MSX1-K139 as a potential therapeutic target. The partial rescue of the cleft palate phenotype suggests that targeting deacetylases or designing K139 stabilizers could represent a novel strategy for early cleft palate intervention. Additionally, the MSX1 acetylation level may serve as a noninvasive prenatal biomarker for cleft palate.

While our study employed a well-established atRA-induced cleft palate model to elucidate a novel pathogenic pathway, it is important to contextualize its scope and limitations. As a potent morphogen, atRA activates broad retinoic acid (RA) signaling, which can pleiotropically influence numerous cellular processes and molecular targets, such as the Wnt and TGF-β, beyond the axis identified here. However, several findings from our data strongly suggest that the SIRT1-MSX1 acetylation–degradation axis is a critical and specific node in this complex teratogenic response. First, the direct manipulation of MSX1 acetylation status—through the deacetylation-mimic K139R mutation—was sufficient to significantly reduce MEPM apoptosis in vitro (Fig. 3D) and partially rescue the cleft phenotype in vivo, even in the continuous presence of atRA (Fig. 6). Conversely, the acetylation-mimic K139Q mutant exacerbated apoptosis (Fig. 3D). This genetic evidence demonstrates that the MSX1 acetylation switch can decisively alter cellular fate independently of, or downstream of, other concurrent RA-mediated effects. Second, our transcriptomic analysis revealed that MSX1 acetylation does not globally alter gene expression (Fig. 4), effectively negating its rapid proteostatic regulation from the potential slow, indirect transcriptional effects of the RA pathway. Therefore, our work delineates a precise post-translational mechanism through which a teratogenic signal (atRA-induced SIRT1 downregulation) is transduced into a key morphogenetic defect (MEPM apoptosis via MSX1 destabilization). Future studies in genetic or other environmental models of cleft palate will be valuable for testing the generalizability of this acetylation-dependent regulatory switch beyond the atRA context.

### Contextualizing acetylation in craniofacial PTM networks

The emerging landscape of craniofacial PTMs encompasses remarkable regulatory sophistication. While SUMOylation governs maxillary prominence fusion [28] and histone methylation directs frontonasal patterning [29], our work establishes acetylation as the master switch controlling palatal shelf competence. This finding complements recent findings that neural crest-specific HDAC3 ablation induces cleft palate through chromatin remodeling [30], suggesting dual epigenetic roles of acetylation, specifically nuclear (histone) acetylation and cytoplasmic (nonhistone) acetylation. Notably, MSX1 acetylation exemplifies a noncanonical degradation mechanism distinct from that of classical acetyl-ubiquitin competition models [31]. Unlike p53, the acetylation of which enhances both its stability and transcriptional activity [32], MSX1 acetylation creates a “silent” loss-of-function state through pure proteostatic regulation—a novel paradigm in transcription factor biology.

### Therapeutic implications and the paradox of partial rescue

Our preclinical rescue experiments revealed promising yet incomplete therapeutic potential: while SIRT1 and MSX1 overexpression rescued the cleft palate phenotype in 29% and 69% of cases, respectively, the K139R mutation primarily reduced cleft severity rather than incidence. This partial efficacy likely stems from developmental stage-specific vulnerability windows missed by interventions administered at E10.5 and compensatory acetylation at secondary sites. These limitations, however, illuminate potential refined strategies, such as the use of small-molecule SIRT1 activators with superior tissue penetration, acetylation-resistant MSX1 variants incorporating multisite mutations, and proteasome timing inhibitors during critical fusion stages. Notably, the strong inverse correlation between MSX1 acetylation and protein levels in embryos with cleft palate suggests that MSX1 acetylation is a quantifiable biomarker for prenatal risk stratification.

### Divergent roles of lysine acetylation in proteostatic regulation

The functional dichotomy of the role of lysine acetylation in protein homeostasis presents a fascinating regulatory paradox. While canonical studies emphasize the role of acetylation in stabilizing proteins by competing with ubiquitination at shared lysine residues (e.g., p53 and E2F1) [33], emerging evidence reveals context-dependent destabilization mechanisms in which lysine acetylation promotes protein degradation, as the acetylation of specific lysine residues results in the recruitment of complexes containing ubiquitin ligase E3 or leads to complex dissociation, increasing the susceptibility of their components to protein degradation [33–36]. Aligning with this noncanonical paradigm, our work demonstrated that similar to HIF1α and T-Ag, lysine acetylation promotes MSX1 protein degradation via the ubiquitination–proteasome pathway [34, 37]. The unique feature of the MSX1 acetylation‒degradation axis is that rapid MSX1 turnover during palatal fusion necessitates acetylation-sensitive regulation, which emphasizes the evolutionary specialization of PTM networks in developmental processes requiring exquisite spatiotemporal control.

### Context-dependent functional diversification of MSX1 in palatogenesis

While previous studies have established the dual role of MSX1 in coordinating the proliferation and apoptosis of progenitor cells before palatogenesis [19, 38, 39], our study involving an atRA-induced cleft palate model revealed a critical window of vulnerability (E13.5–E14.5) where apoptotic priming exceeded compensatory proliferation. At this time, MSX1 may switch from “dual proliferation-apoptosis” in the initial stage of the neural crest process to “single apoptosis” in the fusion stage of the palatal process to ensure precise docking of the palatal shelf. This temporal shift aligns with the “morphogenetic timer” hypothesis, which postulates that key regulators undergo functional repurposing across developmental phases [5]. In addition, the upregulation of TGFβ3 expression by atRA can inhibit the suppression of proliferation caused by MSX1 deficiency [40], resulting in an “apoptosis-sensitive but proliferation tolerant” phenotype.

### Concluding perspectives

This work revealed a previously unrecognized regulatory mechanism through which MSX1, through acetylation, regulates protein stability and cell apoptosis during palate development. Our discovery of the deacetylase SIRT1‒MSX1 acetylation axis not only provides a mechanistic explanation for the incomplete penetrance of MSX1 mutations in cleft palate but also opens avenues for biomarker development. Quantifying MSX1 acetylation levels in amniotic fluid-derived exosomes could enable early prenatal risk stratification. Furthermore, SIRT1 activators or engineered K139-targeted stabilizers may represent novel preventive therapies for high-risk pregnancies. In summary, our findings provide a new direction for further research into the pathogenic mechanisms of cleft palate and offer a new strategy for the diagnosis and prevention of cleft palate. Future efforts should explore acetylation crosstalk with emerging regulatory layers (e.g., liquid‒liquid phase separation [9]) while advancing SIRT1 activators and K139-targeted stabilizers toward clinical application.

## Materials and Methods

### Animals

C57BL/6 mice (8–12 weeks old, 20–25 g) were purchased from Animal Core Facility of Nanjing Medical University, Nanjing, China. Females were mated, and embryonic gestation day of 0.5 (E0.5) was designated for the detection of a positive vaginal plug. At E10.5, pregnant mice were randomly divided into two groups: The mice given a 100 mg/kg body weight of all-trans retinoic acid (atRA) (Macklin, C12056220) by gavage were defined as the atRA group, while those given an equivalent volume of corn oil (Aladdin, C116023) were defined as the control group (Control). The embryos were dissected on E13.5, E14.5, E15.5 and E16.5 under a stereomicroscope. Sample sizes were chosen based on prior pilot experiments and common practice in similar published studies. Each experimental group contained at least *n* = 3 pregnant mice. No samples or animals were excluded from the analysis. All collected data were included. All the animal studies were approved by the Institutional Animal Care and Use Committee at Ninth People’s Hospital, School of Medicine, Shanghai Jiao Tong University (SH9H-2024-A1439-1) and reported in accordance with the ARRIVE guidelines.

### Antibodies

Primary antibodies against the following proteins were used: pH3 (Santa-Cruz, A2971), PCNA (Boster, BM0104), MSX1 (Bioss, bs-8512R), Ac-lysine (Santa-Cruz, sc-32268), SIRT1 (Santa-Cruz, sc-74465), CBP (Santa-Cruz, sc-7300), p300 (Santa-Cruz, sc-48343), GFP (Santa-Cruz, sc-9996), Ubiquitin (Cell Signaling, 3936), β-tubulin (Affinity, T0023) and GAPDH (Affinity, T0004).

### In vivo lentiviral overexpression

The negative control (Lv-Con), mouse Sirt1 (Lv-Sirt1), mouse Msx1 (Lv-Msx1) and mouse Msx1 K139R (Lv-K139R) overexpression lentiviruses were purchased from GenePharma (Shanghai, China). The relevant sequences were obtained from the NCBI Gene Database. Pregnant mice were randomly divided into five groups (*n* = 3 in each group): Control mice injected with Lv-Con, atRA mice injected with Lv-Con, atRA injected with Lv-Sirt1, atRA mice injected with Lv-Msx1, and atRA mice injected with Lv-K139R. All the lentivirus were intraperitoneally injected (3 × 10^8^ TU/mL in 100 μL PBS) into pregnant mice at E10.5. This time point was selected on the basis of preliminary optimization: earlier administration at E8.5 and E9.5 resulted in substantially increased embryonic mortality (100% and 69.1%, data not shown), whereas E10.5 ensured viable pregnancies and sufficient transgene expression before the critical palatal fusion window.

### Histology analyses

Collected fetal heads were fixed with 4% paraformaldehyde (PFA) overnight, dehydrated, embedded in paraffin, and sliced into 4-µm-thick serial sections using an automated rotary microtome (Leica, RM2265, Germany). To observe of the general morphology, the slices were stained with hematoxylin and eosin (H&E) according to standard protocols.

### Immunofluorescence staining

For paraffin section staining, the slices were deparaffinized and rehydrated stepwise. For zebrafish embryo and cell staining, the tissues and cells were fixed with 4% PFA for 30 min. The samples were then permeabilized with 0.5% Triton X-100 for 20 min at room temperature and blocked in goat serum at 37 °C for 60 min, followed by incubation with the indicated primary antibodies at 4 °C overnight. The samples were counterstained with specific secondary antibodies and DAPI staining solution (Beyotime, C1005) for nuclear staining. Images of the samples were captured by microscopy (Leica Microsystems, DM4000, Wetzlar) under 488 nm and 594 nm laser lines. Protein expression and colocalization levels were quantified by ImageJ software.

### Detection of cell apoptosis

Terminal deoxynucleotidyl transferase dUTP nick-end labeling (TUNEL) staining assay and flow cytometry were performed for cell apoptosis analysis. For TUNEL staining, tissues and cells were collected and stained with an I*n Situ* Cell Death Kit (Roche, 12156792910) and DAPI according to the guide of the manufacturer’s instructions. For flow cytometry, cells were collected by centrifugation and stained with Annexin-V-Fluorescein isothiocyanate (annexin-V-FITC) and propidium iodide (PI). The stained cells were analyzed by a flow cytometry (BD Biosciences, FACSCalibur, USA). FlowJo V6.2 software was used to analyze the data.

### Cell culture

The palatal shelves of E13.5 embryos were dissected under a surgical microscope. The tissues were subsequently washed three times with PBS containing 1% penicillin-streptomycin (P/S) (NCM Biotech, C100C5) and then incubated with 0.25% trypsin (Gibco, 25200072) for 15 min at 37 °C to dissociate individual mouse embryonic palatal mesenchymal (MEPM) cells. The action of trypsin was inhibited by fetal bovine serum (FBS) (VivaCell, C04001-050). Primary cultures of MEPM cells were made in Dulbecco’s Modified Eagle’s Medium (DMEM)/Ham’s Medium F12 (1:1) (Gibco, C11330500BT) supplemented with 10% FBS and 1% P/S. HEK293T cells (RRID: CVCL_0063) were purchased from the American Type Culture Collection (ATCC, CRL-11268). Cells were routinely tested for mycoplasma contamination and authenticated by STR profiling, and grown in DMEM media (VivaCell, C3110-0500) supplemented with 10% FBS and 1% P/S. The cells were all seeded in 100-mm-diameter cell-culture dishes at 37 °C in a humidified atmosphere with 5% CO_2_. The media were replaced every other day, and the confluent cells were subcultured at a ratio of 1:3. For some experiments, cells were treated with nicotinamide (NAM) (Aladdin, N105042) (20 mM, 6 h), trichostatin A (TSA) (Beyotime, S1893) (500 nM, 6 h), EX527 (MCE, HY-15452) (10 µM, 24 h), MG132 (MCE, HY-13259) (10 µM, 4 h) and CHX (Cayman, 14126) (50 mM, 0–12 h).

### Plasmids

A GFP-tagged MSX1 expression plasmid was generated by subcloning full-length MSX1 cDNA into pmEGFP-N2 mutated by pEGFP-N2 (Clontech). The MBP-MSX1-mEGFP plasmid was generated by PCR and subcloned into the pET-MBP-3C vector. Flag-tagged *homo sapiens* ubiquitin B (UBB) was purchased from iGeneBIO (Guangzhou, China). The MSX1 K139R, K139Q, K237R and K243R mutations were generated by site-directed mutagenesis using PCR cloning. FLAG-SIRT1 was purchased from Viraltherapy Technologies (Wuhan, China). GFP-tagged MSX1 truncation mutants (Δ43-79, Δ79-172, and Δ172-239) were generated by GFP-MSX1 using PCR cloning. All the constructs were verified by DNA sequencing. The plasmids were transfected with ExFect Transfection Reagent (Vazyme, T101-01) following the manufacturer’s instructions.

### shRNAs and siRNAs

Short hairpin RNA (shRNA) and small interfering RNA (siRNA) were purchased from GenePharma (Shanghai, China). The sequences were as follows:

**Table:** 

**Name**	**Sequence**
shNC	5’- TTCTCCGAACGTGTCACGT -3’
shMSX1	5’- GGCCAAGAGATTTATCCGT -3’
siNC	5’- UUCUCCGAACGUGUCACGUTT -3’
siSIRT1	5’- CCAUGAAGUGCCUCAAAUATT -3’

The transfections of shRNA and siRNA were performed with Lipofectamine 2000 (Thermo Fisher, 11668019) following the manufacturer’s instructions. The knockdown efficiency was verified by quantitative real-time PCR (qRT‒PCR).

### qRT‒PCR

Total RNA was extracted from cells or tissues using a FastPure Cell/Tissue Total RNA Isolation Kit (Vazyme, RC112) according to the manufacturer’s instructions. The total RNA was subjected to synthesize complementary DNA synthesis, and PCR was subsequently conducted in a qRT‒PCR instrument (Applied Biosystems, ABI7300, USA) with the following thermocycler settings: 95 °C for 10 min; 40 cycles of 95 °C for 10 s, 60 °C for 15 s, and 72 °C for 15 s. The primers used were shown in Supplementary Table 1. The data were normalized and analyzed with the GraphPad Prism 8 software.

### Protein extraction and western blotting

The tissues and cells were lysed with RIPA buffer (Beyotime, P0013B) supplemented with 1% phenylmethanesulfonylfluoride (PMSF) (Beyotime, ST505) at 4 °C and sonicated on ice, and then centrifuged at 12,000 rpm for 20 min to collect the supernatant. The protein solution was diluted in 5 × SDS buffer (Beyotime, P0015) and boiled for 5 min. The protein was loaded in equal amounts, separated via 10% SDS‒PAGE and then transferred to 0.22 µm PVDF membranes (Millipore, ISEQ00010), which were subsequently blocked with 5% nonfat milk and incubated with the indicated primary antibodies at 4 °C overnight. After being washed with Tris-buffered saline containing 1% tween (TBST), the membranes were incubated with an HRP-conjugated secondary antibody for 60 min at room temperature. The membranes were visualized by chemiluminescence (Tanon, Tanon 5200, China).

### Immunoprecipitation (IP) and Co-IP

A cell lysis reagent specifically for IP (Beyotime, P0013J) containing 1% PMSF was used to lyse the cells and collect the proteins. The primary antibodies or control IgG were first conjugated with Protein A + G Magnetic Beads (Beyotime, T2108) in a vertical rotating incubator at 4 °C for 8 h, followed by the incubation with cell lysates for another 8 h. The immune-precipitates were subsequently washed with PBS containing 0.1% Tween 20 (PBST) for three times. After eluting in 5 × SDS buffer and boiling for 5 min, the samples were analyzed by western blotting.

### Recombinant protein expression and purification

Plasmids containing MBP-tagged genes were transformed into *E. coli* BL21 (DE3)-Rosetta cells (TIANGEN, CB108) and incubated in LB media supplemented with 100 mg/ml ampicillin (Sangon Biotech, A610029) in a shaker at 37 °C and 180 rpm. Once the OD600 value reached 0.6–0.8, 0.1 mM IPTG (Yeason, 10902ES08) was added to induce protein expression and the induction was maintained at 18 °C at 120 rpm for an additional 18 h. The cells were harvested and ultrasonicated in lysis buffer (50 mM Tris-HCl pH 7.5, 500 mM NaCl, and 1 mM PMSF) with setting parameters of 80% of 300 W, 5 s on and 10 s off for 1.5 h. The supernatant containing the dissolved fusion protein was first purified with Ni-NTA Agarose (QIAGEN, 163026181), and then further purified using Superdex 200 Increase 10/300 GL with AKTA pure. These proteins were flash frozen in liquid nitrogen, and then stored in 150 mM Tris-HCl (pH 7.5), 500 mM NaCl, 1 mM DTT, and 10% glycerol at −80 °C. The purity of the proteins was determined by SDS–PAGE followed by Coomassie blue staining. The protein concentration was determined by a BCA protein quantification kit (Vazyme, E112-01) with Nanodrop measurement for OD 562 nm. Recombinant His6-SIRT1 protein was purchased from MCE (#HY-P71596).

### Transcriptome Sequencing Analysis

RNA sequencing was performed on MEPM across four experimental groups: empty vector control (Vector), wild-type MSX1 overexpression (MSX1), deacetylation-mimetic mutant (K139R), and acetylation-mimetic mutant (K139Q). Each group included three biological replicates (*n* = 3). Total RNA was extracted using TRIzol reagent (Thermo Fisher, 15596026). Sequencing was conducted on an Illumina NovaSeq 6000 platform (150 bp paired-end reads), with a target depth of 40 million reads per sample. The quality control of the raw data was performed using FastQC (v0.11.9), followed by adapter trimming and low-quality base removal (Phred score < 20) via Trimmomatic (v0.39). Gene expression quantification was performed with featureCounts (v2.0.3). Differentially expressed genes (DEGs) were identified using DESeq2 (v1.34.0), with thresholds set at |log2(fold change)| ≥ 2 and FDR-adjusted *p* < 0.05. Functional enrichment analysis for GO terms and KEGG pathways was conducted using clusterProfiler (v4.4.4).

### Statistical Analysis

All experiments were performed independently for at least three times. All of the data were analyzed with Student’s t-test to compare the means between two groups. The data are expressed as the means ± standard deviations. *P* < 0.05 was considered to indicate a statistically significant difference.

## Supplementary information

Supplemental material

Original western blots

## Data Availability

The data that support the findings of this study are available in the supplementary material of this article. RNA-sequencing data have been deposited in the Gene Expression Omnibus (GEO) under accession number GSE316532.
