# Supplementary material for: Acetylation-triggered degradation of MSX1 impairs palatal development
Source: Cell Death Discov. 2026 Mar 19;12:156. doi: 10.1038/s41420-026-03018-w (PMC13039415; doi:10.1038/s41420-026-03018-w)
Supplement: Supplementary file 1 — Supplemental material [file 41420_2026_3018_MOESM1_ESM.docx]

***Supplementary Material***

**Acetylation-Triggered Degradation of MSX1 Impairs Palatal Development**

Li Meng^#1^, Jiawen You^#2^, Zhongyin Zhang^3^, Yucheng Jiang^3^, Yulan Liu^1^, Mingliang Zhou*^4^, Junqing Ma*^5^, Xinquan Jiang*^1, 4^

^1^ Department of Prosthodontics, Shanghai Ninth People’ s Hospital, Shanghai Jiao Tong University School of Medicine, College of Stomatology, Shanghai Jiao Tong University, National Center for Stomatology, National Clinical Research Center for Oral Diseases, Shanghai Key Laboratory of Stomatology, Shanghai Engineering Research Center of Advanced Dental Technology and Materials, Shanghai 200125, China.

^2^ Stomatological Hospital affiliated Suzhou Vocational Health College, Suzhou 215002, China.

^3^ State Key Laboratory Cultivation Base of Research, Prevention and Treatment for Oral Diseases, Nanjing Medical University, Nanjing 210029, China.

^4^ Shanghai Stomatological Hospital, Fudan University, Shanghai 201102, China.

^5^ Savaid Stomatology School, Hangzhou Medical College, Hangzhou 311399, China; Hangzhou Stamotological Hospital (Zijingang Campus), Hangzhou 310030, China.

**^#^**These authors contributed equally to this work.

***Correspondence**

**Corresponding Author:** Xinquan Jiang, E-mail: xinquanjiang@aliyun.com.

Junqing Ma, E-mail: majunq@163.com

Mingliang Zhou, E-mail: mingliangzhou@aliyun.com.

**Keywords:** MSX1, acetylation, embryonic palatal mesenchymal, apoptosis, cleft palate

**Figure 1**

**
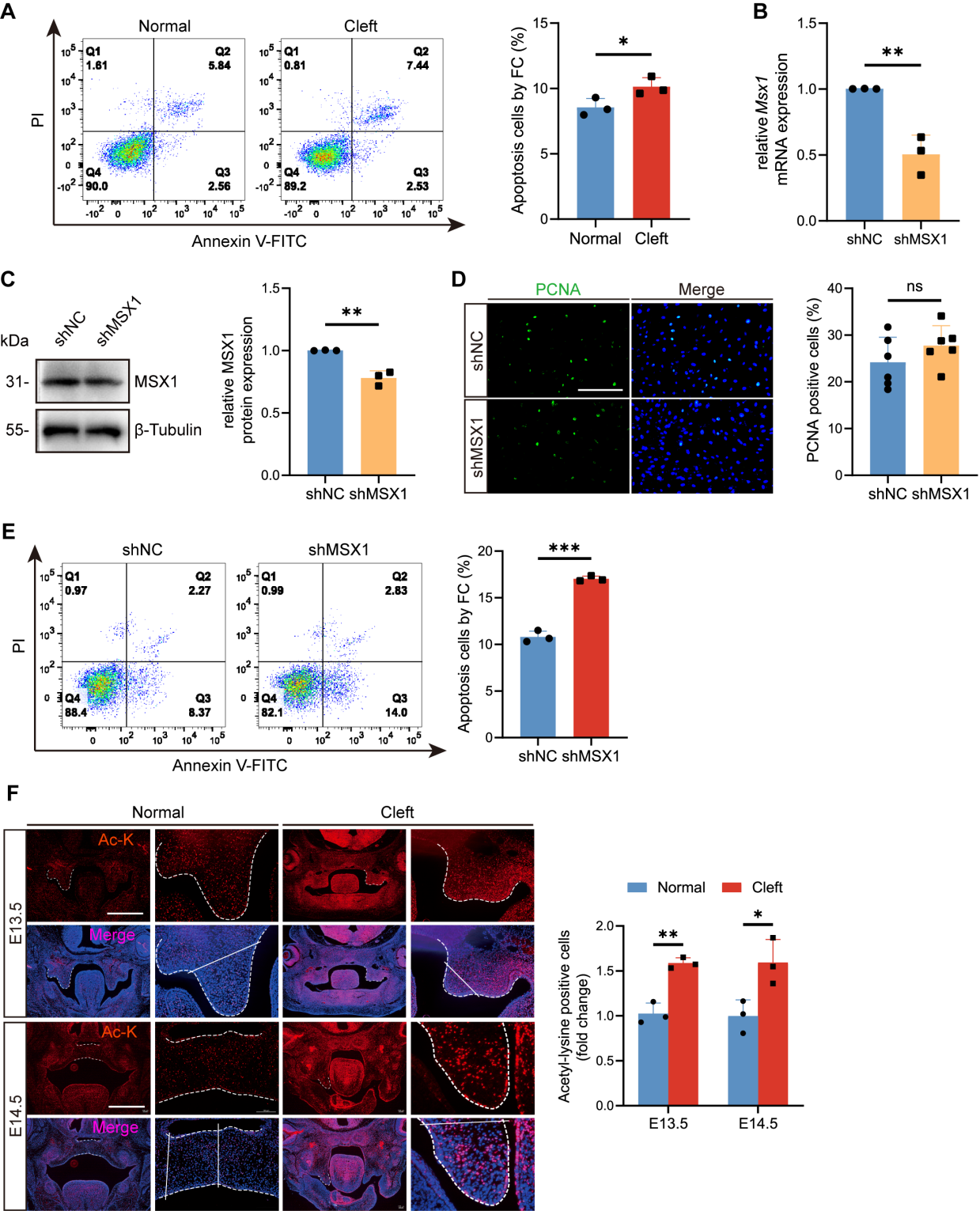
**

**Fig 1. AtRA induces cleft palate through aberrant MEPM cell apoptosis and MSX1 acetylation**

**(A)** Flow cytometry analysis of MEPM cells extracted from E14.5 embryos. n=3. **(B)** Efficiency of shRNA-mediated MSX1 knockdown in MEPM cells measured by qRT-PCR. n=3. **(C)** Efficiency of shRNA-mediated MSX1 knockdown in MEPM cells measured by western blot and quantitative analysis of MSX1 protein level. n=3. **(D)** PCNA immunostaining (green) of MEPM cells transfected with shNC and shMSX1, and quantitative analysis. Bars, 200 µm. n=6. **(E)** Flow cytometry analysis of cell apoptosis in MEPM cells transfected with shNC or shMSX1..n=3. **(F)** Immunofluorescent staining of acetyl-lysine (red) of palatal shelves of E13.5 and E14.5 mice in Normal and Cleft groups. Bars, 200 µm. n=3. Ac-K, Ac-lysine. Data are presented as the mean ± standard deviation (Mean ± SD). ns, not significant, *p<0.05, **p<0.01, ***p<0.001.

**Figure 2**

**
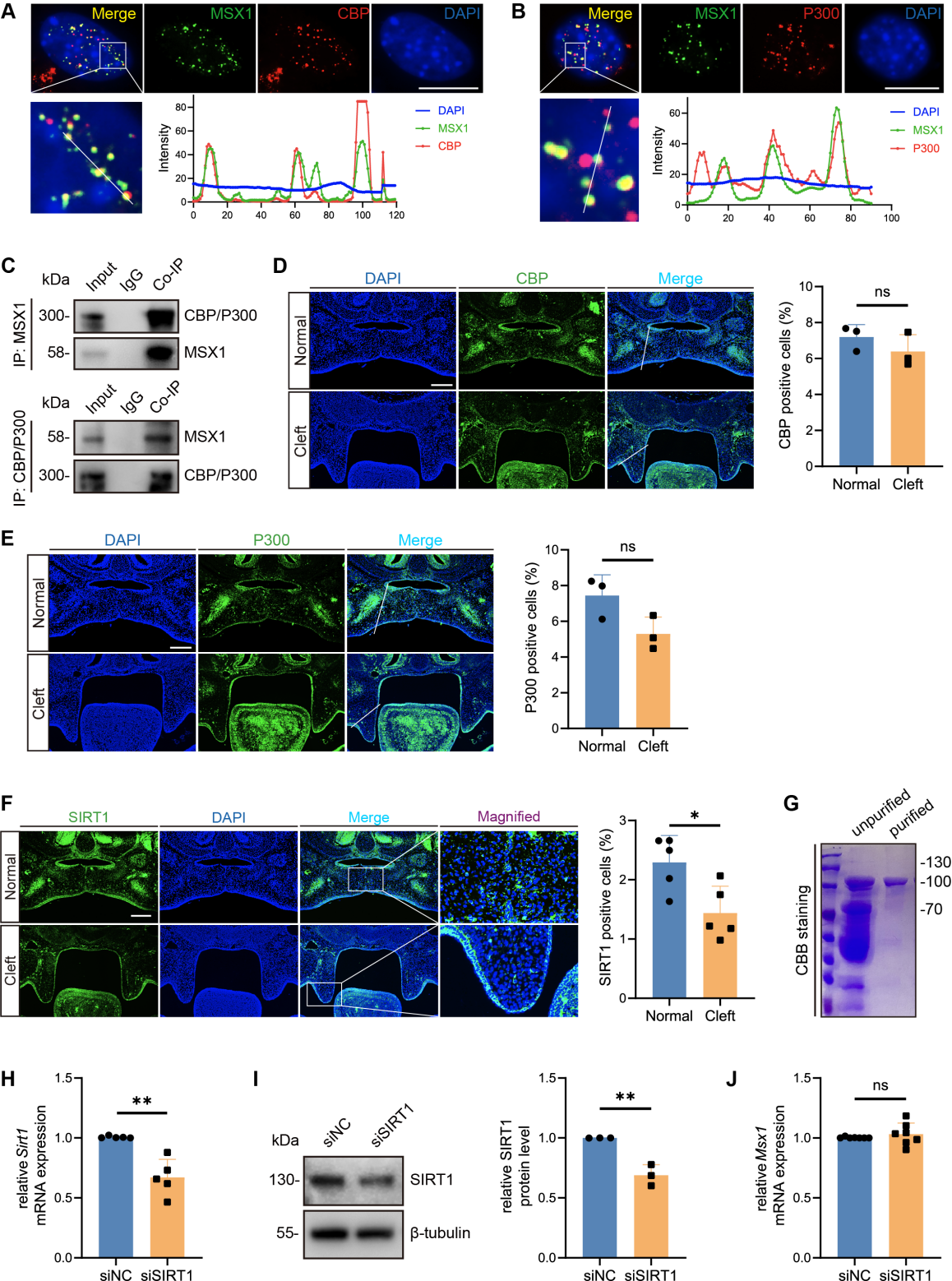
**

**Fig 2. SIRT1 governs MSX1 deacetylation at K139**

**(A)** Immunofluorescence colocalization staining of MSX1 and CBP. Bars, 200 µm. **(B)** Immunofluorescence colocalization staining of MSX1 and P300. Bars, 200 µm. **(C)** Co-IP analysis of MSX1 with CBP/P300. **(D)** Immunofluorescent staining of CBP (green) of palatal shelves from E14.5 embryos and quantitative analysis. Bars, 200 µm. n=3. **(E)** Immunofluorescent staining of P300 (green) of palatal shelves from E14.5 embryos and quantitative analysis. Bars, 200 µm. n=3. **(F)** Immunofluorescence staining of SIRT1 (green) of palatal shelves from E14.5 embryos and quantitative analysis. Bars, 200 µm. n=5. **(G)** Coomassie brilliant blue (CBB) staining of the MBP-MSX1-GFP recombinant proteins. **(H)** Efficiency of siRNA-mediated SIRT1 knockdown in MEPM cells measured by qRT-PCR. n=5. **(I)** Efficiency of siRNA-mediated SIRT1 knockdown in MEPM cells measured by western blot and quantitative analysis. n=3. **(J)** MSX1 mRNA expression measured by qRT-PCR in siNC and siSIRT1 groups. n=7. Data are presented as the mean ± standard deviation (Mean ± SD). ns, not significant, *p<0.05, **p<0.01.

**Figure 3**

**
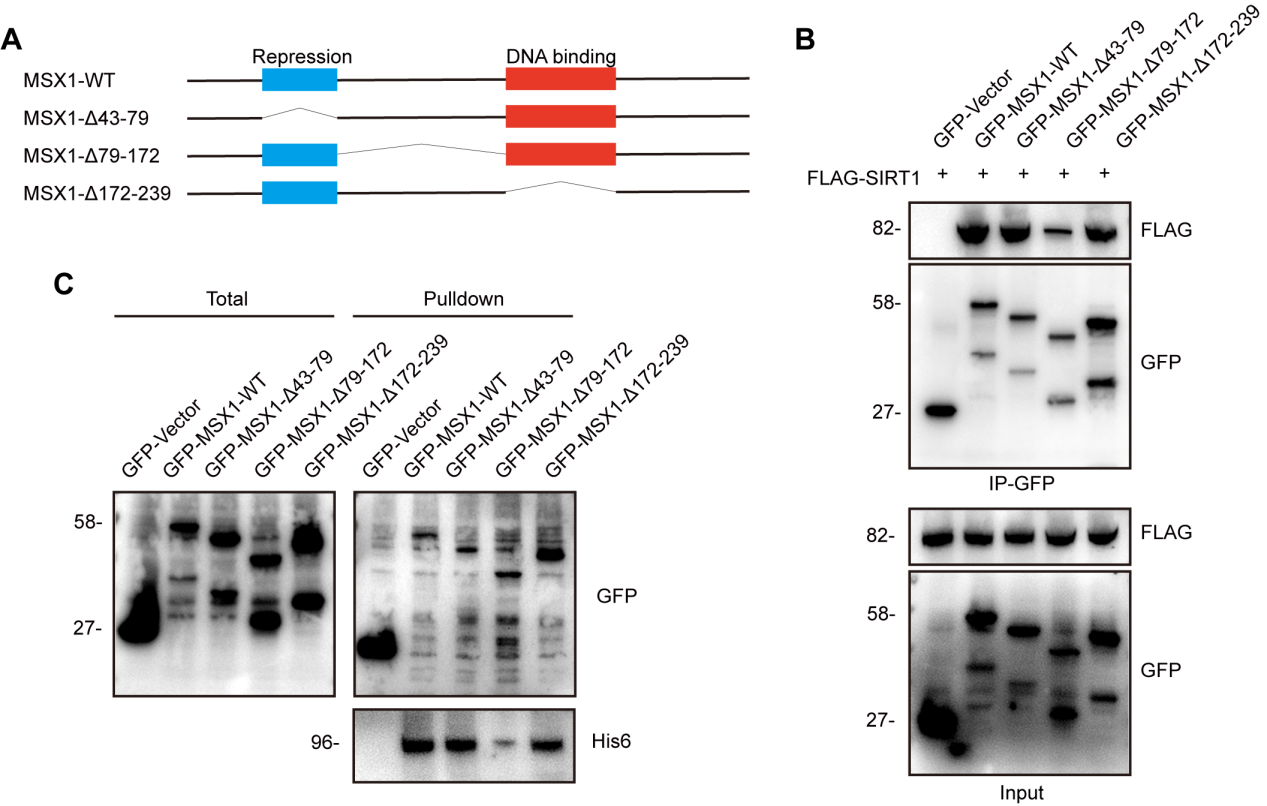
**

**Fig 3. MSX1 interacts with SIRT1 at specific segment where acetylation occurs.**

**(A)** Diagram of full-length and truncated GFP-MSX1 proteins. **(B)** Co-IP analysis of FLAG-SIRT1 with GFP- tagged MSX1 wild-type (WT) and MSX1 truncation mutants (Δ43-79, Δ79-172, and Δ172-239). **(C)** GFP pulldown analysis of His6-SIRT1 with GFP- tagged MSX1 wild-type (WT) and MSX1 truncation mutants (Δ43-79, Δ79-172, and Δ172-239).

**Figure 4**

**
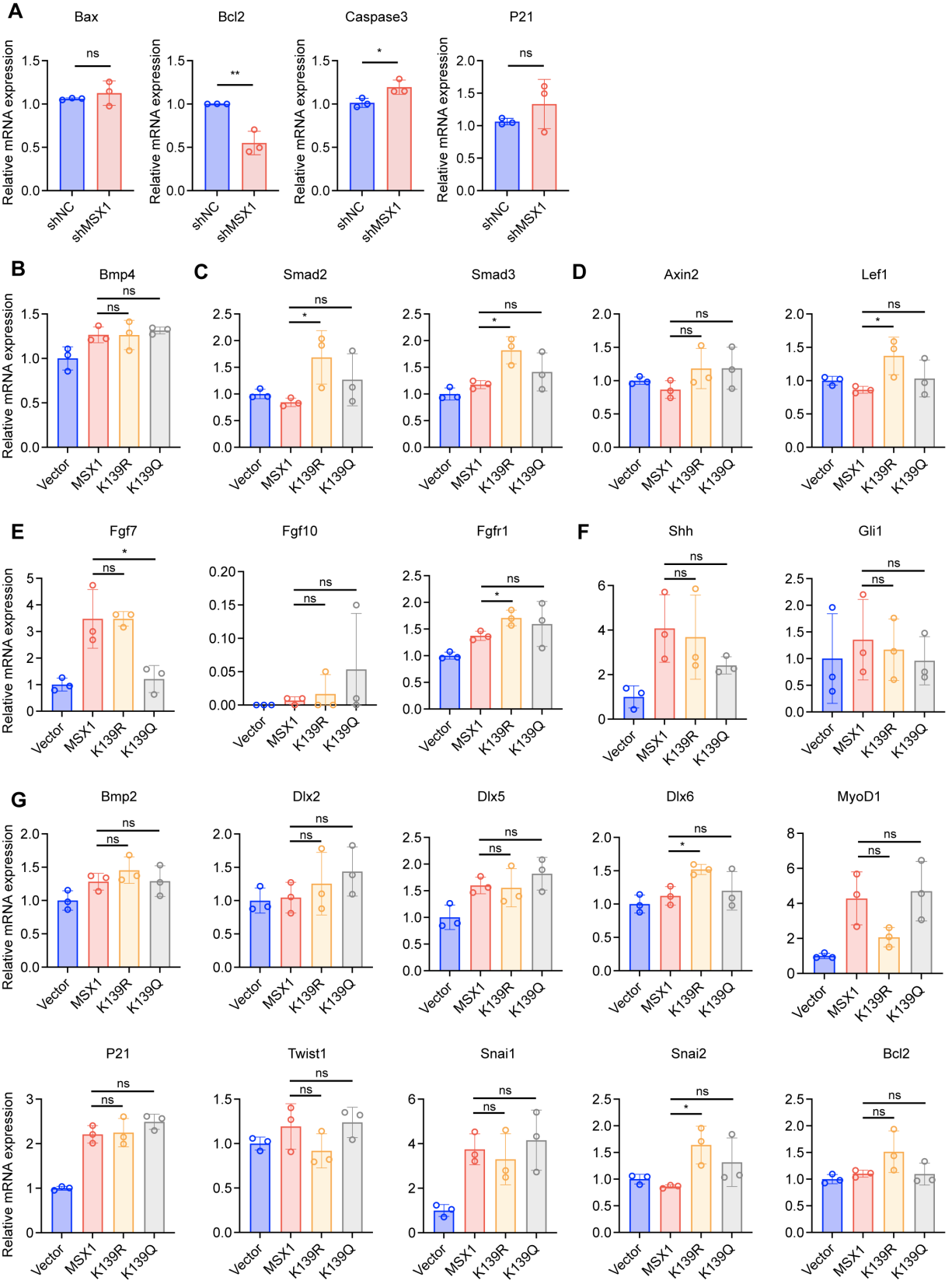
**

**Fig 4. Lysine acetylation of MSX1 promotes proteasomal degradation**

1. qRT-PCR analysis of apoptosis-related genes in total RNA extracted from MEPM. n=3. (B) qRT-PCR analysis of *BMP* signaling pathway in total RNA extracted from MEPM. n=3. **(C)** qRT-PCR analysis of TGFβ signaling pathway in total RNA extracted from MEPM. n=3. **(D)** qRT-PCR analysis of WNT/β-catenin signaling pathway in total RNA extracted from MEPM. n=3. **(E)** qRT-PCR analysis of FGF signaling pathway in total RNA extracted from MEPM. n=3. **(F)** qRT-PCR analysis of SHH signaling pathway in total RNA extracted from MEPM. n=3. **(G)** qRT-PCR analysis of MSX1 transcriptional targets in total RNA extracted from MEPM. n=3. Data are presented as the mean ± standard deviation (Mean ± SD). ns, not significant, *p<0.05, **p<0.01.

**Figure 5**

**
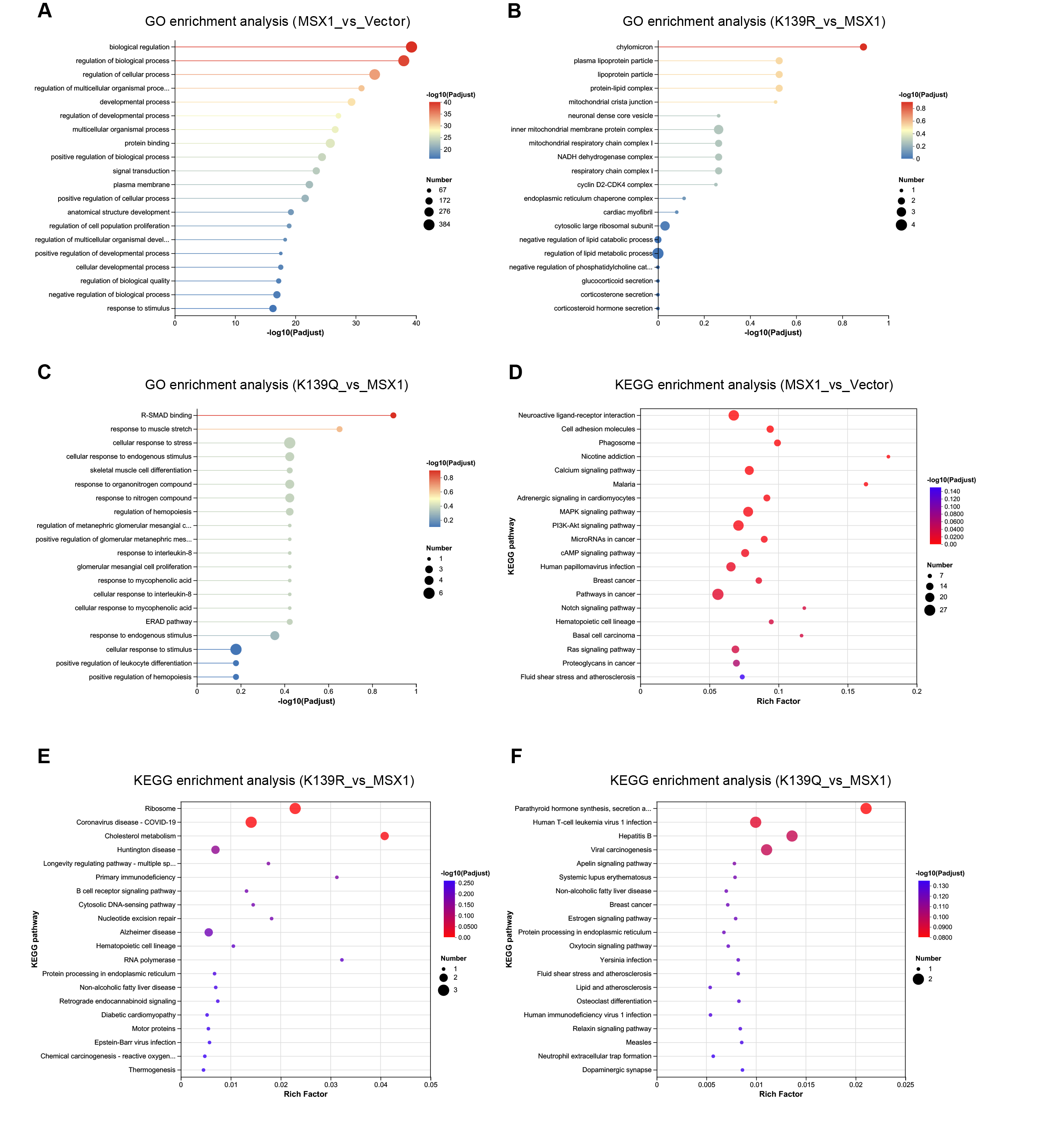
**

**Fig 5. Lysine acetylation of MSX1 promotes proteasomal degradation**

**(A-C)** Gene Ontology Enrichment Analysis (GO) of DEGs between MSX1 and Vector groups, K139R and MSX1 groups, K139Q and MSX1 groups. **(D-E)** KEGG pathways analysis of DEGs between MSX1 and Vector groups, K139R and MSX1 groups, K139Q and MSX1 groups.

**Figure 6**

**
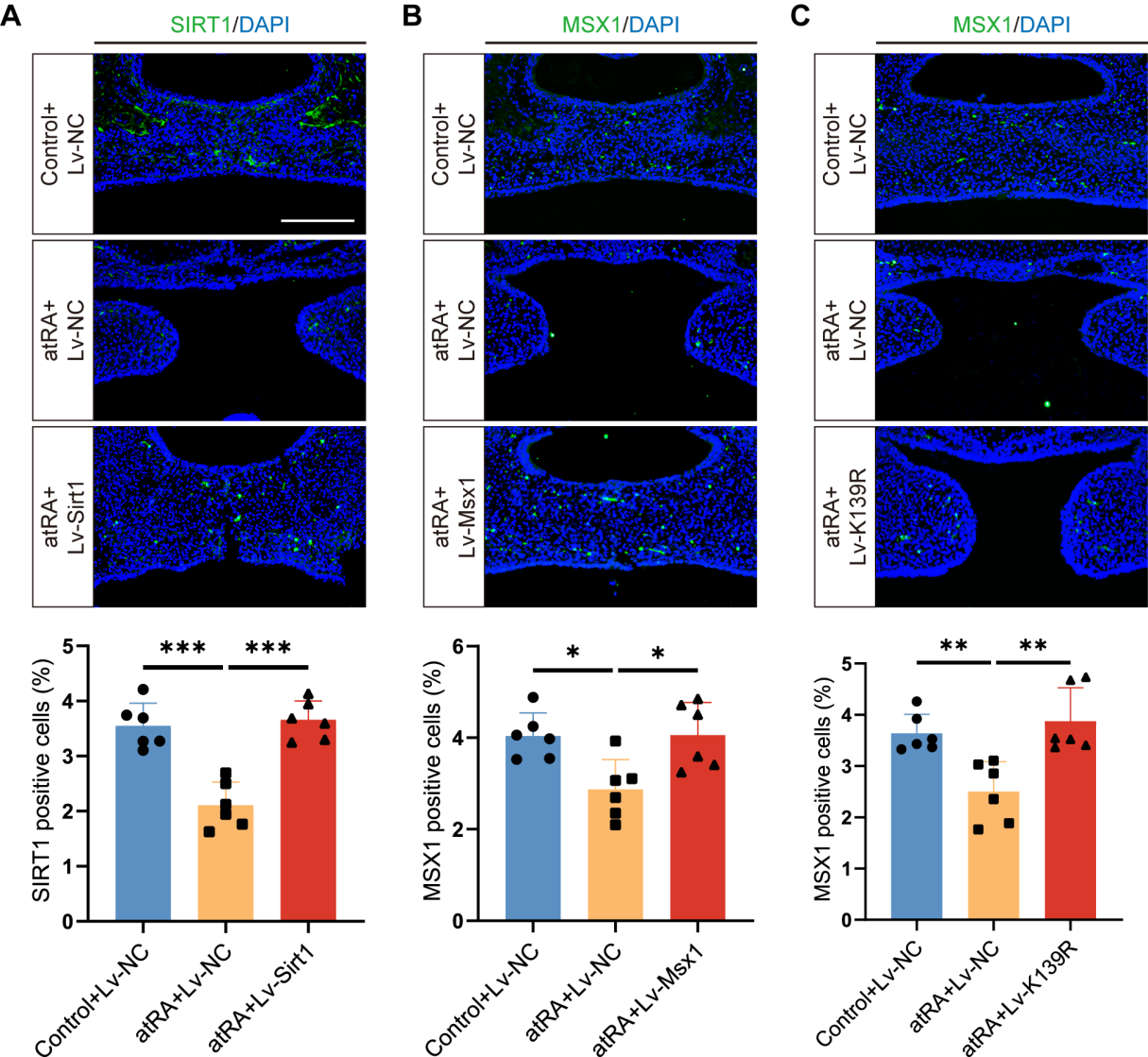
**

**Fig 6. Reducing MSX1 lysine acetylation partially rescues atRA-induced cleft palate**

**(A-C)** Immunofluorescent staining and quantification of SIRT1 or MSX1 (green) in palatal shelves of lentivirus infected embryos at E15.5. Nuclei were stained with DAPI. Bars, 200 µm. n=6. Data are presented as the mean ± standard (Mean ± SD). ns, not significant, *p<0.05, **p<0.01, ***p<0.001.

**Table 1**

The primers used for qRT‒PCR.

| **Name** | **Sequence** | |
| --- | --- | --- |
| Gapdh | F | 5’-ACCACAGTCCATGCCATCAC-3’ |
|  | R | 5’-TCCACCACCCTGTTGCTGTA-3’ |
| Msx1 | F | 5’-TCATGGCCGATCACAGGAAG-3’ |
|  | R | 5’-GGAGTCCTCCGACTGAGAAATG-3’ |
| Sirt1 | F | 5’-GCTGACGACTTCGACGACG-3’ |
|  | R | 5’-TCGGTCAACAGGAGGTTGTCT-3’ |
| Bmp4 | F | 5’-GGAACACCACGAGCAAGACA-3’ |
|  | R | 5’-CTCCGGGTTATGTTCTCCAG-3’ |
| Smad2 | F | 5’-TGAAGCCATCCACAGCTTCT-3’ |
|  | R | 5’-CGGACACCTCAATGGTGTTC-3’ |
| Smad3 | F | 5’-CCTGGCTGTGTGAGAAAGGA-3’ |
|  | R | 5’-TGCATAGACTGGTCCTGGTG-3’ |
| Axin2 | F | 5’-TGACTCTCCTTCCAGATCCC-3’ |
|  | R | 5’-TGCCCACACTAGGCTGACA-3’ |
| Lef1 | F | 5’-CCTACCCAGCCAGACTCAAA-3’ |
|  | R | 5’-GGCTGCATCTCCCTCTTTCT-3’ |
| Fgf7 | F | 5’-CTGGCTGTCATGGAGAACCT-3’ |
|  | R | 5’-TCCAGGGTCTTGGTAGTTGG-3’ |
| Fgf10 | F | 5’-GCTGGAGAAACCAAGCAAGA-3’ |
|  | R | 5’-CTTGGCACATCCAAACACAC-3’ |
| Fgfr1 | F | 5’-CAGCCGTGTGTTTCTGTGAC-3’ |
|  | R | 5’-GCTGGTAGGTGATGCTGATG-3’ |
| Shh | F | 5’-CGGAGATTGAGAAGAGGTGG-3’ |
|  | R | 5’-CAGGTGAGGAAGTCGCTGTA-3’ |
| Gli1 | F | 5’-AAGCCAAGTTATTGGTGCGG-3’ |
|  | R | 5’-GCTGGGATGTTAAGGGCATT-3’ |
| Bmp2 | F | 5’-CGAAACGAGTGGGAAACCTC-3’ |
|  | R | 5’-GCTGTAGTGCGGCAACAC-3’ |
| Dlx2 | F | 5’-CCCTACCCAGTGCTCAAGAC-3’ |
|  | R | 5’-CTGCTGCCTCTGTGTGTTCT-3’ |
| MyoD1 | F | 5’-CACTACAGCGGCGACTCC-3’ |
|  | R | 5’-GCTCCACTATGCTGGACAGG-3’ |
| P21 | F | 5’-CCGTGGACAGTGAGCAGTT-3’ |
|  | R | 5’-CATGAGCGATGGGAACTTC-3’ |
| Bcl2 | F | 5’-GCTACCGTCGTGACTTCGC-3’ |
|  | R | 5’-TCCACAAAGGCATCCCAGCC-3’ |
| Caspase 3 | F | 5’-CTCTGGTTTTCGGTGGGTGT-3’ |
|  | R | 5’-CTTCCATGTATGATCTTTGGTTCC-3’ |
| Bax | F | 5’-AAACTGGTGCTCAAGGCCC-3’ |
|  | R | 5’-AAAGTAGGAGAGGAGGCCGT-3’ |
