# Supplementary material for: Acetylation-triggered degradation of MSX1 impairs palatal development
Source: Cell Death Discov. 2026 Mar 19;12:156. doi: 10.1038/s41420-026-03018-w (PMC13039415; doi:10.1038/s41420-026-03018-w)

Figure. 1H

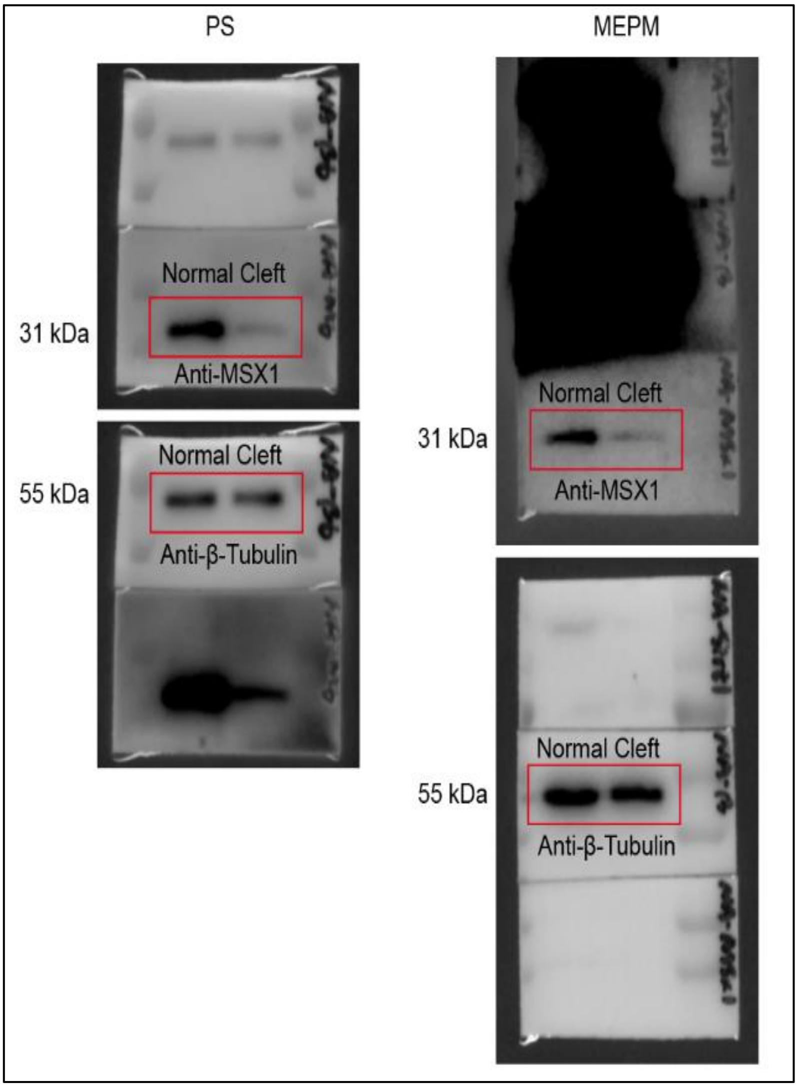

Figure. 1M

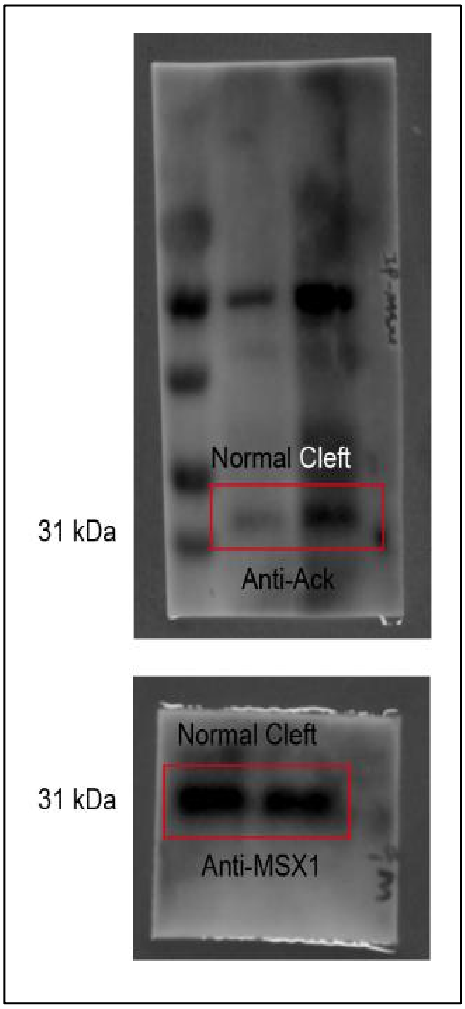

Figure. 2A

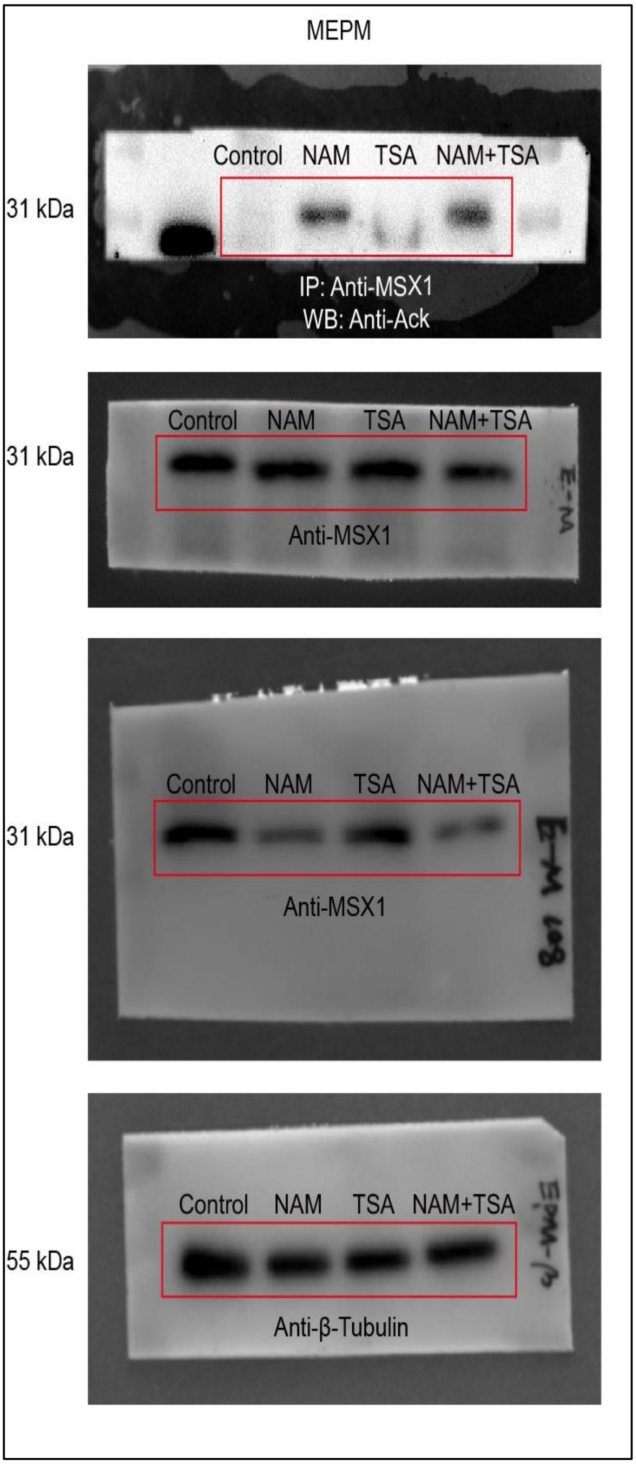

Figure. 2B

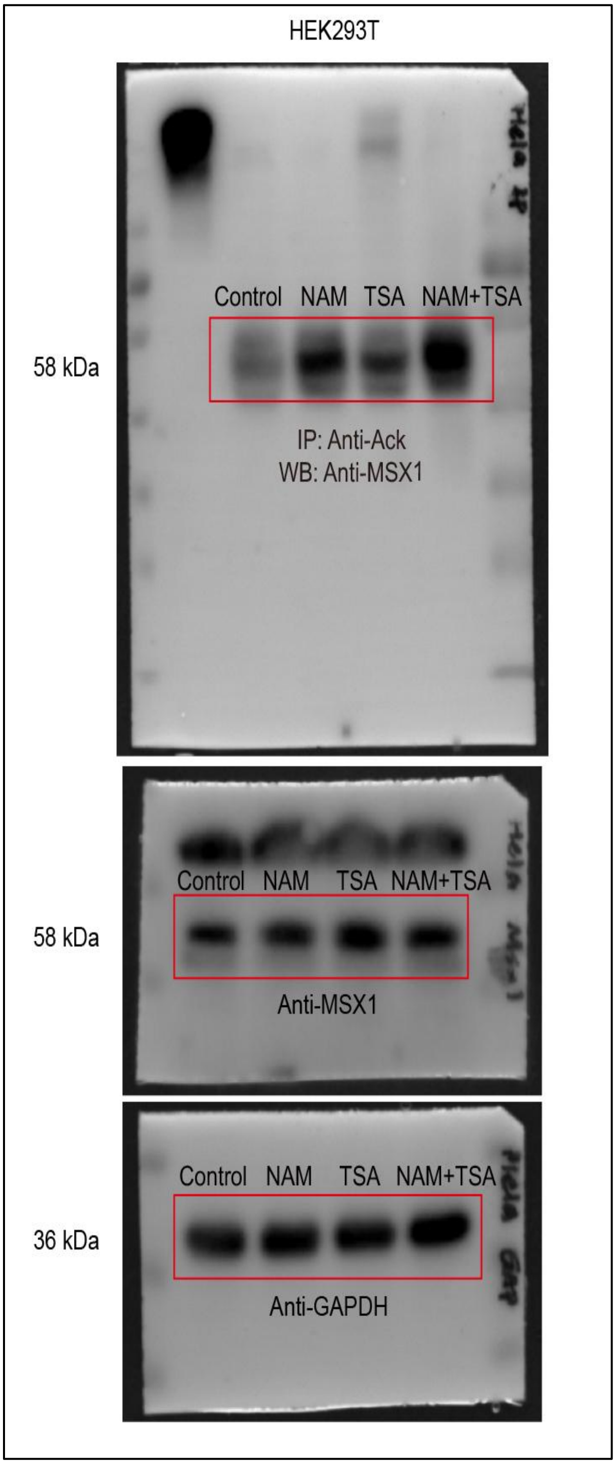

Figure. 2E

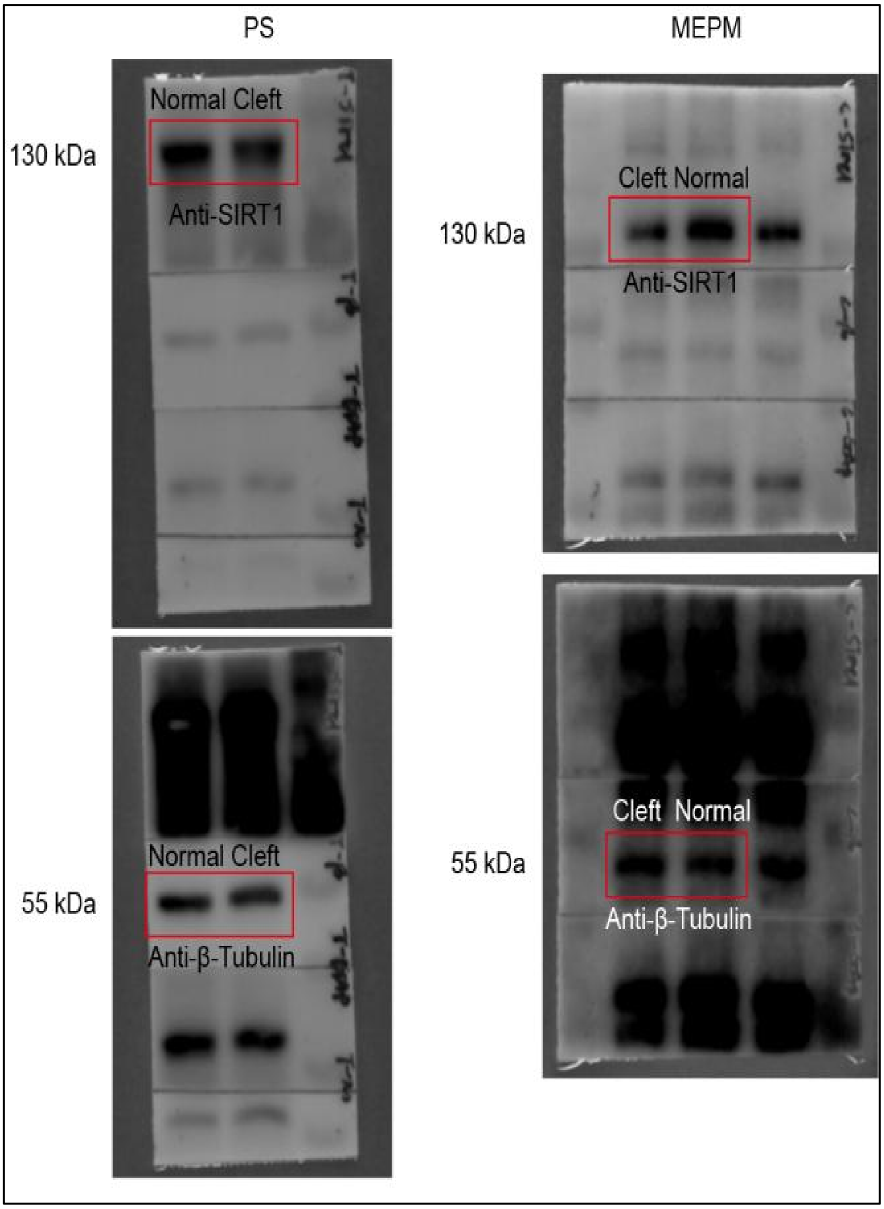
PS
MEPM
Normal Cleft
130 kDa
Anti-SIRT1
Cleft Normal
130 kDa
Anti-SIRT1
Normal Cleft
55 kDa
Anti-β-Tubulin
Cleft Normal
55 kDa
Anti-β-Tubulin

Figure. 2G

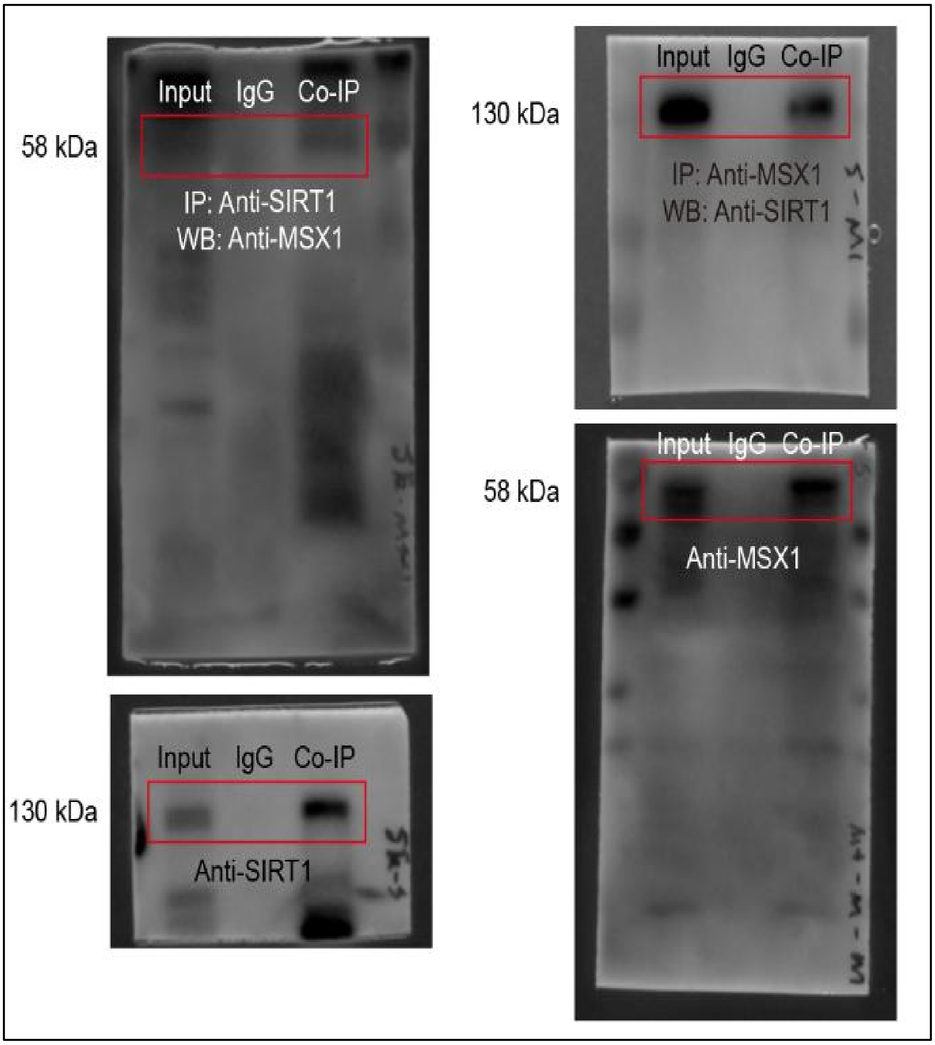
58 kDa
Input IgG Co-IP
IP: Anti-SIRT1
WB: Anti-MSX1
130 kDa
Input IgG Co-IP
Anti-SIRT1
130 kDa
Input IgG Co-IP
IP: Anti-MSX1
WB: Anti-SIRT1
58 kDa
Input IgG Co-IP
Anti-MSX1

Figure. 2H

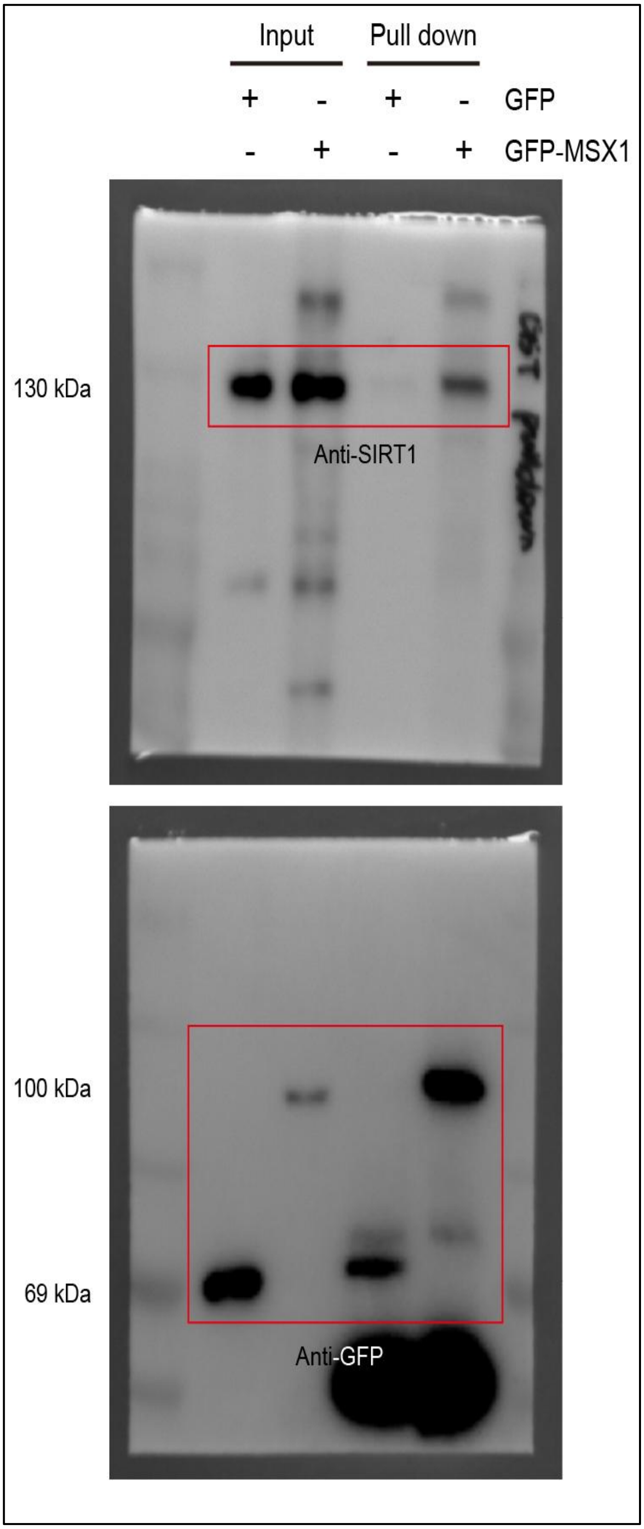
Input
Pull down
+ - + - GFP
- + - + GFP-MSX1
130 kDa
Anti-SIRT1
100 kDa
69 kDa
Anti-GFP

Figure. 2I

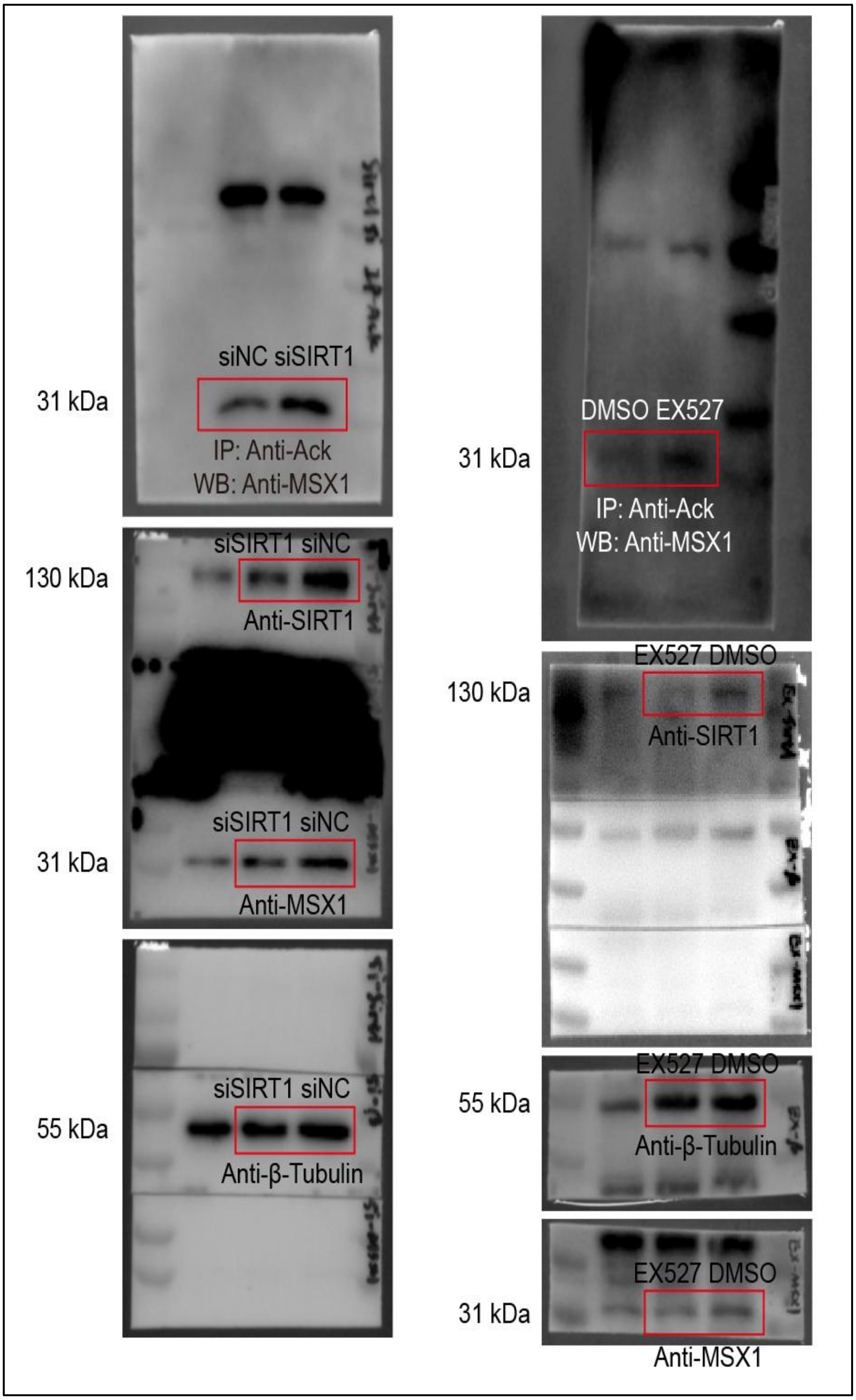

siNC siSIRT1
31 kDa
IP: Anti-Ack
WB: Anti-MSX1
siSIRT1 siNC
130 kDa
Anti-SIRT1
siSIRT1 siNC
31 kDa
Anti-MSX1
siSIRT1 siNC
55 kDa
Anti-β-Tubulin
DMSO EX527
31 kDa
IP: Anti-Ack
WB: Anti-MSX1
EX527 DMSO
130 kDa
Anti-SIRT1
EX527 DMSO
55 kDa
Anti-β-Tubulin
EX527 DMSO
31 kDa
Anti-MSX1

Figure. 2K

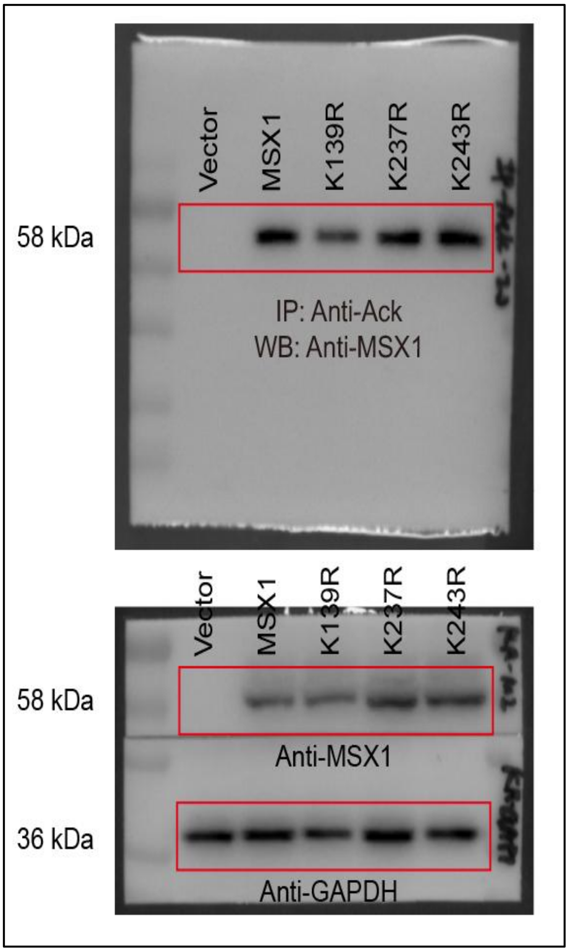

Figure. 5A

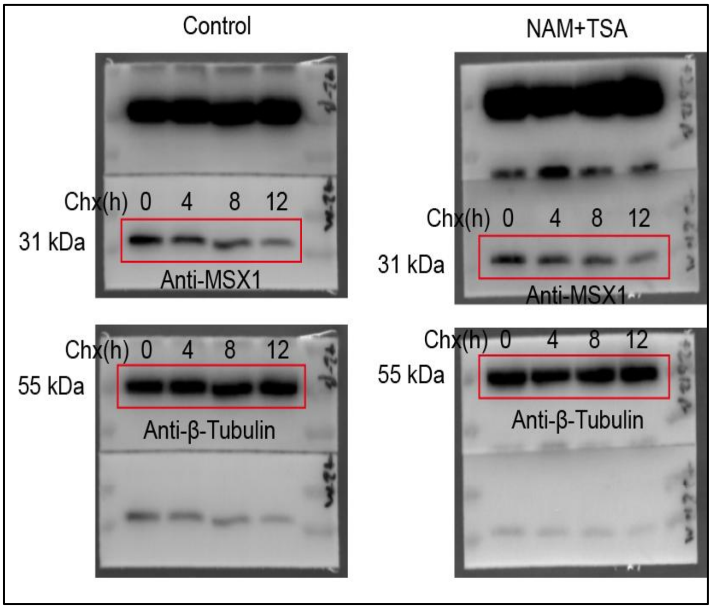

Figure. 5B

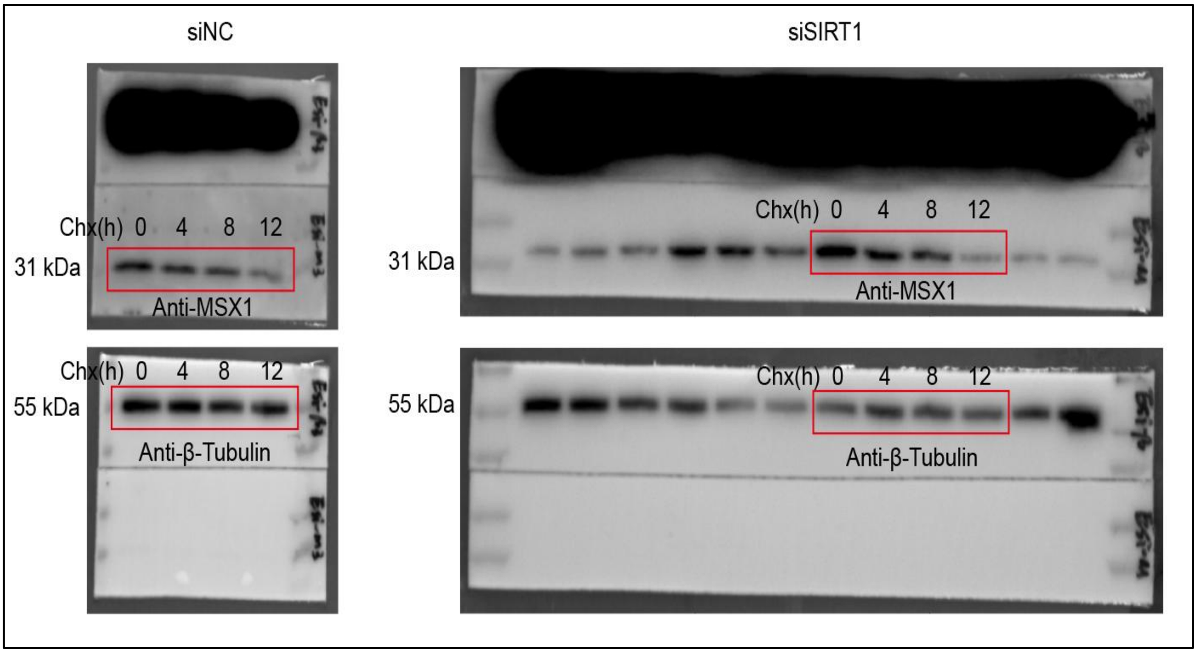

Figure. 5C

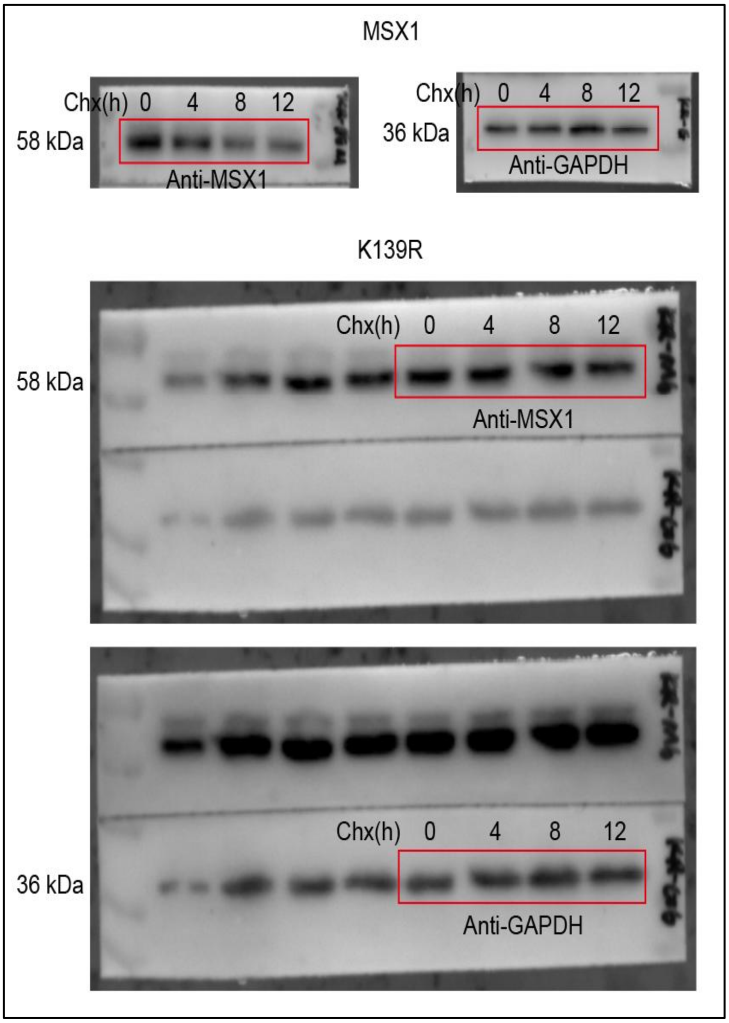

Figure. 5D

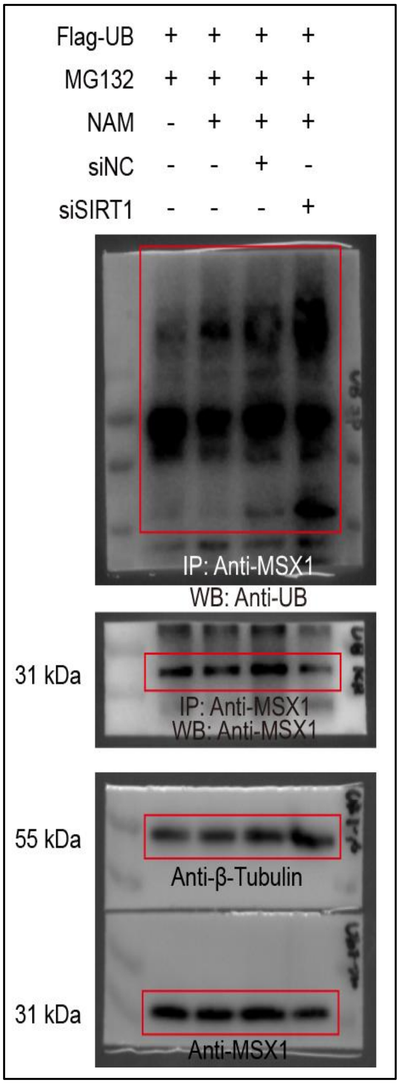

Figure. 5E

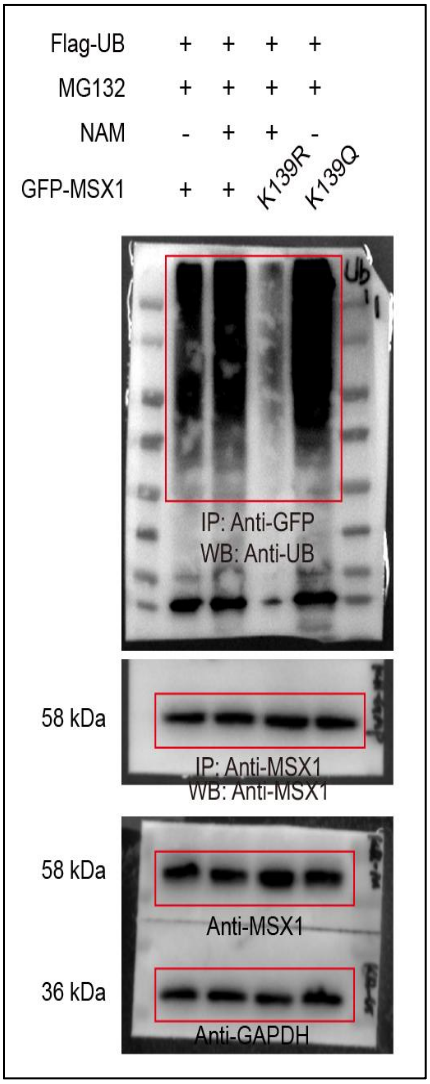

Supplementary Figure. 1C

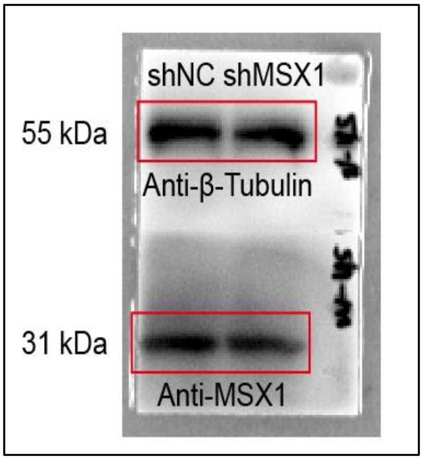

Supplementary Figure. 2C

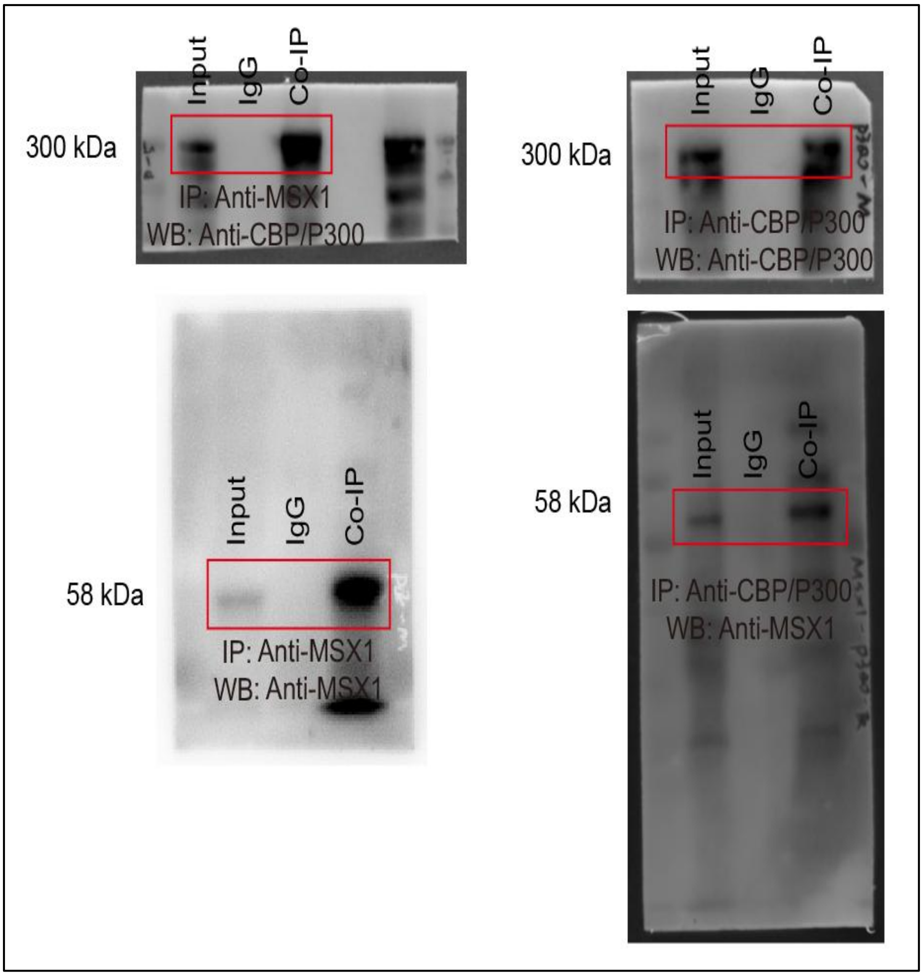

Supplementary Figure. 2G

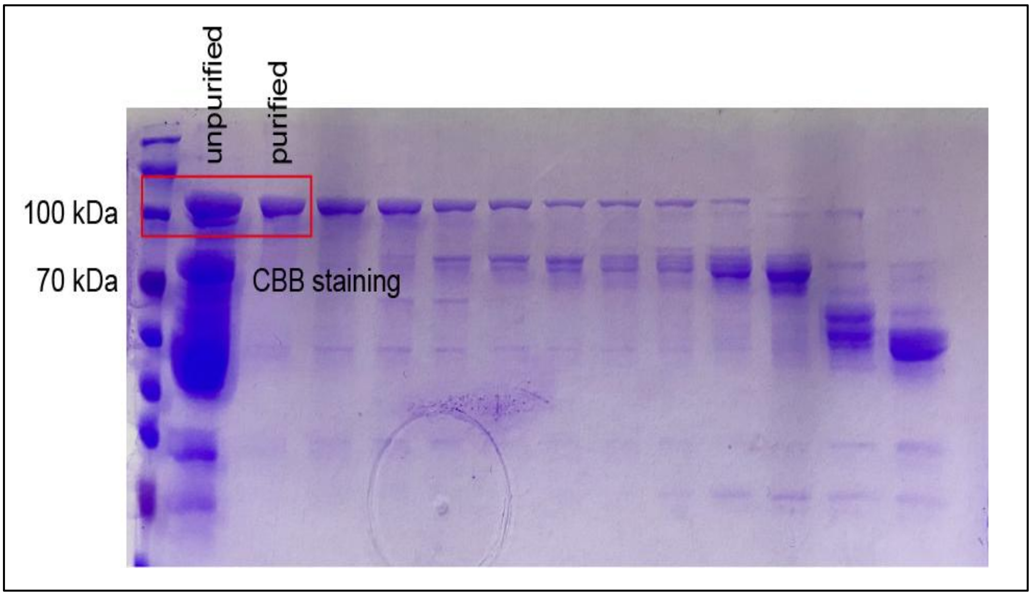

Supplementary Figure. 2I

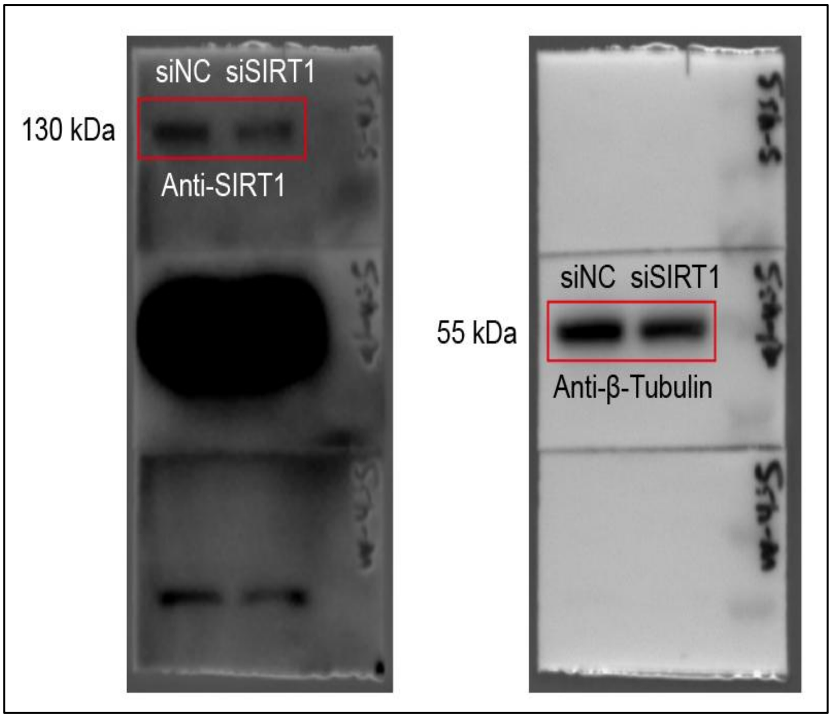

Supplementary Figure. 3B

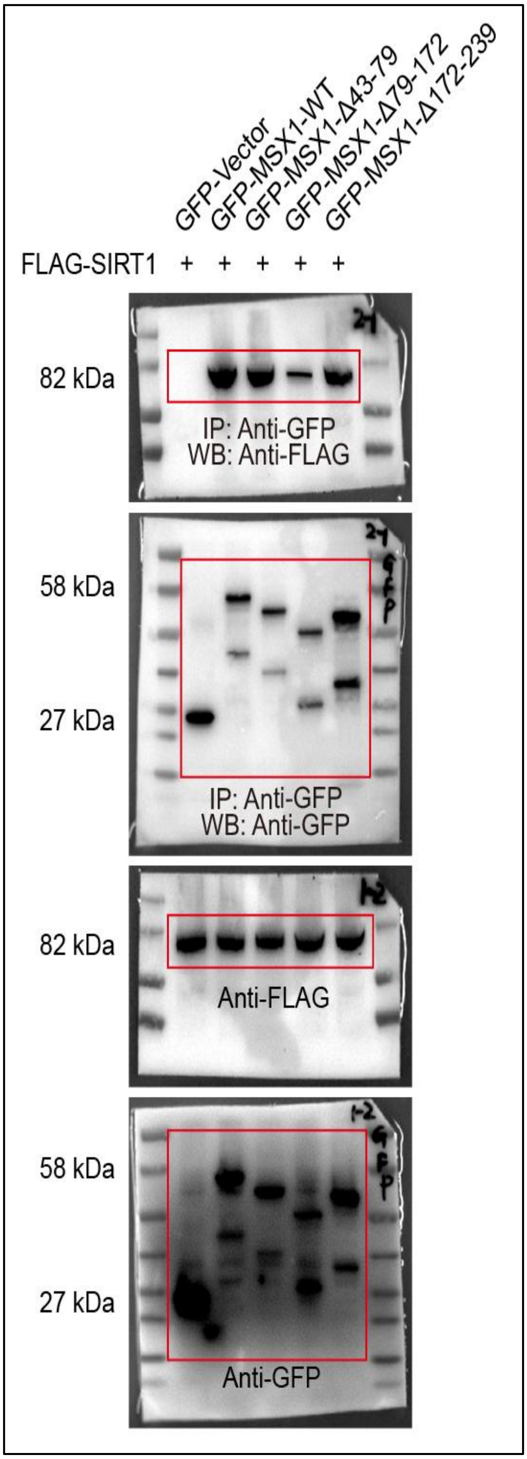

Supplementary Figure. 3C

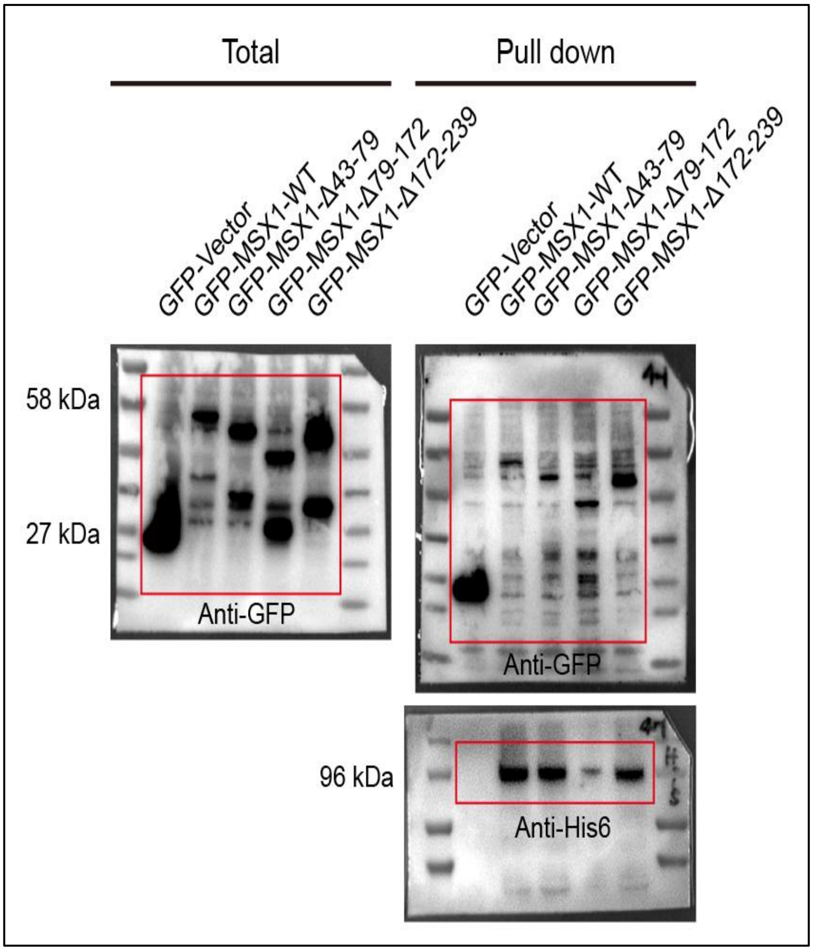

Supplement: Supplementary file 2 — Original western blots [file 41420_2026_3018_MOESM2_ESM.pdf]
